# ZBP1 causes inflammation by inducing RIPK3-mediated necroptosis and RIPK1 kinase activity-independent apoptosis

**DOI:** 10.1038/s41418-024-01321-6

**Published:** 2024-06-07

**Authors:** Lioba Koerner, Laurens Wachsmuth, Snehlata Kumari, Robin Schwarzer, Theresa Wagner, Huipeng Jiao, Manolis Pasparakis

**Affiliations:** 1 Institute for Genetics, University of Cologne, 50674 Cologne, Germany; 2 Cologne Excellence Cluster on Cellular Stress Responses in Aging-Associated Diseases (CECAD), University of Cologne, 50931 Cologne, Germany; 3 Center for Molecular Medicine (CMMC), Medical Faculty and University Hospital Cologne, University of Cologne, 50931 Cologne, Germany; 4 Present Address: Frazer Institute, The University of Queensland, Faculty of Medicine, Brisbane, QLD Australia; 5 Present Address: Genentech Inc, South San Francisco, CA USA; 6 Present Address: Life Sciences Institute, Zhejiang University, Hangzhou, China

**Keywords:** Cell death and immune response, Chronic inflammation

## Abstract

Z-DNA binding protein 1 (ZBP1) has important functions in anti-viral immunity and in the regulation of inflammatory responses. ZBP1 induces necroptosis by directly engaging and activating RIPK3, however, the mechanisms by which ZBP1 induces inflammation and in particular the role of RIPK1 and the contribution of cell death-independent signaling remain elusive. Here we show that ZBP1 causes skin inflammation by inducing RIPK3-mediated necroptosis and RIPK1-caspase-8-mediated apoptosis in keratinocytes. ZBP1 induced TNFR1-independent skin inflammation in mice with epidermis-specific ablation of FADD by triggering keratinocyte necroptosis. Moreover, transgenic expression of C-terminally truncated constitutively active ZBP1 (ZBP1ca) in mouse epidermis caused skin inflammation that was only partially inhibited by abrogation of RIPK3-MLKL-dependent necroptosis and fully prevented by combined deficiency in MLKL and caspase-8. Importantly, ZBP1ca induced caspase-8-mediated skin inflammation by RHIM-dependent but kinase activity-independent RIPK1 signaling. Furthermore, ZBP1ca-induced inflammatory cytokine production in the skin was completely prevented by combined inhibition of apoptosis and necroptosis arguing against a cell death-independent pro-inflammatory function of ZBP1. Collectively, these results showed that ZBP1 induces inflammation by activating necroptosis and RIPK1 kinase activity-independent apoptosis.

## Introduction

Z-DNA binding protein 1 (ZBP1, also known as DLM1 and DAI) has emerged as a potent mediator of cell death, inflammation and immunity. ZBP1 contains two N-terminal Zα domains that specifically bind to Z-form nucleic acids, DNA and double stranded (ds)RNA with an alternative left-handed double helix structure [1–3]. In addition, ZBP1 contains three C-terminal receptor interacting protein (RIP) homotypic interaction motifs (RHIMs) that facilitate its interaction with other RHIM-containing proteins, including RIPK1, RIPK3 and TIR-domain-containing adapter-inducing interferon-β (TRIF) [4, 5]. ZBP1 has been shown to bind and activate RIPK3, resulting in RIPK3 auto-phosphorylation and the subsequent phosphorylation of mixed lineage kinase-like (MLKL), which translocates to the plasma membrane causing necroptosis [6, 7]. Moreover, ZBP1 was reported to activate inflammatory signaling by interacting with RIPK1 and RIPK3 to induce activation of nuclear factor kappa B (NF-κB)-dependent gene transcription [4, 5]. Furthermore, ZBP1 plays an important role in response to infection with certain viruses, including influenza, vaccinia and murine cytomegalovirus, where it senses viral Z-RNA and induces RIPK3-mediated necroptosis and FADD-caspase-8-mediated apoptosis to restrict viral replication [8, 9].

Studies in genetic mouse models identified ZBP1 as a potent inducer of inflammation in the absence of viral infection. Mice with epidermal keratinocyte-specific ablation of RIPK1 (RIPK1^E-KO^) develop severe skin inflammation mediated by RIPK3-MLKL-dependent keratinocyte necroptosis, which is strongly suppressed by ZBP1 deficiency but only partially ameliorated by TNFR1 knockout [6, 10]. In addition, mutation of the RIPK1 RHIM in mice (*Ripk1*^*mR/mR*^) causes perinatal lethality that is dependent on ZBP1-RIPK3-MLKL-mediated necroptosis [6, 7]. These studies showed that RIPK1 acts via its RHIM to counteract activation of ZBP1 and the induction of necroptosis and inflammation. ZBP1 also plays an important role in the pathogenesis of intestinal inflammation in mice with intestinal epithelial cell (IEC)-specific ablation of Fas associated with death domain (FADD), an adapter that is essential for activation of caspase-8 downstream of death receptors. FADD^IEC-KO^ mice develop colitis mediated by RIPK3-MLKL-dependent necroptosis, as well as ileitis that is largely dependent on necroptosis and to a lesser extent on gasdermin D-mediated cell death [11]. Ablation of ZBP1 or TNFR1 alone could not inhibit ileitis in FADD^IEC-KO^ mice, however, combined ablation of both ZBP1 and TNFR1 strongly suppressed the pathology revealing a functional redundancy between these two proteins [11]. In contrast, loss of either ZBP1 or TNFR1 could strongly suppress colitis development in FADD^IEC-KO^ mice, showing that in this tissue both proteins are required to induce the disease [11]. Importantly, mutation of the ZBP1 Zα domains prevented skin inflammation in RIPK1^E-KO^ mice and perinatal lethality in *Ripk1*^*mR/mR*^ mice, as well as intestinal inflammation in FADD^E-KO^ mice [12, 13], arguing that ZBP1 is activated by sensing endogenous Z-nucleic acids via its Zα domains and induces necroptosis and inflammation. ZBP1 was also shown to play an important role in driving intestinal inflammation in mice with IEC-specific knockout of SETDB1, a histone methyltransferase mediating the trimethylation of histone H3 at lysine 9 [14]. Moreover, ZBP1 was recently reported to mediate heatstroke-induced pathology in a RIPK3-dependent manner [15]. Furthermore, ADAR1 mutations were shown to trigger ZBP1-mediated cell death and interferon responses that contribute to the resulting pathology and anti-tumor immunity [16–19]. Collectively, these studies showed that ZBP1 causes inflammation and disease in vivo by activating RIPK3, however, whether ZBP1 can also induce RIPK1-mediated cell death and inflammatory pathology remains unclear.

Here, we investigated the mechanisms by which ZBP1 induces inflammation in vivo. We show that ZBP1 mediates TNFR1-independent inflammation in FADD^E-KO^ mice by inducing RIPK3-MLKL-dependent necroptosis. In addition, using a newly developed genetic mouse model, we show that ZBP1 acts via its first RHIM to activate RIPK3-MLKL-mediated necroptosis and, importantly, to induce RIPK1 kinase activity-independent caspase-8-mediated apoptosis in keratinocytes. Moreover, our results provide experimental evidence that ZBP1 induces skin inflammation exclusively by triggering cell death arguing against a role of ZBP1 in inducing cell death-independent inflammatory signaling.

## Materials and methods

### Mice

K14-Cre [20], *Fadd*^*fl/fl*^ [21], *Tnfr1*^*fl/fl*^ [22], *Zbp1*^−/−^ [13] *Caspase-8*^*fl/fl*^ [23], *Ripk1*^*D138N/D138N*^ [24], and *Ripk1*^*mR/mR*^ [6] mice were described previously. *R26*^*LSL.ZBP1ca*^, *Ripk3*^*mR/mR*^, *Mlkl*^−/−^ and *Mlkl*^*AA/AA*^ mice are described in this study. All mice were maintained on a C57BL/6N background and mouse studies and immunohistochemical analysis were performed in a blinded fashion. For all experiments, littermates carrying the loxP-flanked alleles but not K14-Cre recombinase were used as controls. Female and male animals of the indicated genotypes were randomly assigned to groups. For comparability, the same ZBP1ca^E-het^ mice were used in all graphs.

Animals were maintained at the SPF animal facility of the CECAD Research Center, University of Cologne, at 22 °C (±2 °C), a relative humidity of 55% (±5%) in individually ventilated cages under a 12-h light cycle. Mice were given access to a sterilized commercial diet (Ssniff Spezialdiäten GmbH) and given water ad libitum. Animals requiring medical attention were provided with appropriate care and sacrificed when reaching pre-determined criteria of disease severity. No other exclusion criteria existed. Based on our previous experience on skin disease mouse models we aimed to at least analyze 10 animals per group. No specific method of randomization had been used to select animals.

### Cell culture

Cell lines were cultured in DMEM-Dulbecco’s Modified Eagle Medium (Thermo Fisher, #41965-039) supplemented with 10% FCS, 1% P/S, 1% L-Glutamate and 1 mM Sodium Pyruvate (Thermo Fisher Scientific, #11360).

### Generation of stable cell lines via lentiviral transduction

For lentiviral production, HEK293T cells were transfected with pCW-sFLAG-mZBP1 (10 µg), pCW-sFLAG-ZBP1ca-mZα (10 µg) or pCW-sFLAG-ZBP1ca (10 µg) and psPAX2 (5 µg) (Addgene, #12260) and pMD2.G (5 µg) (Addgene #12259). Plasmids were mixed with 500 µl 0.25 M CaCl_2_ and 500 µl 2xHBS and incubated for 5 min at RT. The solution was added to HEK293T cells, which were fed with new medium prior to transfection. After 1 day, medium was replaced with fresh medium and after 2 and 3 days the supernatant containing the virus was collected. For transduction of immortalized mouse embryonic fibroblasts (iMEFs), 200,000 cells were incubated with 1.5 ml fresh medium, 1.5 ml viral supernatant and 8 µg/ml Polybrene (Sigma-Aldrich, #H9268) for 24 h. After 1 day, the virus-containing medium was replaced with new medium containing 2 µg/ml puromycin. After 2 days, puromycin-containing medium was removed and cell pools were used for experiments. All cell lines were regularly checked for mycoplasma contamination.

### Cell death assays

iMEFs were plated 24 h before stimulation in a 96 well plate (Sigma-Aldrich, CLS3340-50EA, 1 × 10^4^ cells/well). Cells were stimulated with combinations out of Doxycycline (Sigma-Aldrich D9891), Emricasan (5 µM, Selleckchem, #S7775), Necrostatin 1 s (20 µM, BioCat, 2263-5-BV) or GSK’872 (3 µM Millipore). Cell death was measured by Draq7 (0.15 µM, Cell Signaling, 7406) incorporation, normalized to Draq5 (40845, Cell Signaling) staining and analyzed using the IncuCyte bioimaging platform (Sartorius).

### Immunohistochemical analysis

For Hematoxylin & Eosin (H&E) staining, skin samples were fixed in 4% paraformaldehyde for 24 h, embedded in paraffin andcut in 3–5 µm sections. Paraffin sections were de-paraffinized using xylene and re-hydrated with ethanol. Sections were stained for 2 min in hematoxylin, 15 min differentiated in tap water and 1 min incubated in eosin. Stained sections were de-hydrated with ethanol and fixed in xylene. Epidermal thickness was analyzed by measuring epidermal thickness in four optical fields per sections with four measurements per field.

For cleaved caspase-3 and ZBP1 immunostaining, paraffin-embedded sections were re-hydrated with xylene and ethanol. Heat induced antigen retrieval was performed and endogenous peroxidase was blocked in peroxidase buffer. Endogenous biotin was blocked with avidin (Vector, no. SP-2001) and sections were incubated with primary a-cleaved Caspase-3 antibody (Cell Signaling, 9661) and biotin (Vector, no. SP-2001) overnight at 4 °C. For visualization, the ABC Kit Vectastain Elite (Vector Laboratories) and DAB substrate (DAKO and Vector Laboratories) were utilized. Sections were counterstained with hematoxylin. CC3 positive cells were counted manually in five optical fields per section (×20 magnification). Determination of CC3 positive cells, measurement of epidermal thickness and histologic assessment was performed blindly. For the ZBP1 staining, sections were treated as described above but without pre-treatment of avidin or incubation with biotin. After blocking of endogenous peroxidase, sections were blocked for 30 min with Superblock T20 and incubated overnight with a-ZBP1 antibody (Adipogen, clone Zippy-1, AG-20B-0010-C100) at 4 °C. Sections were washed 3x with PBS-T, incubated with the ImPRESS HRP Universal Antibody for 30 min at RT, washed 3x with PBS-T and visualized with DAB substrate (DAKO and Vector Laboratories)

For TUNEL staining, paraffin-embedded sections were re-hydrated using xylene and ethanol. The TUNEL assay kit (ab206386, abcam) was used and the staining was carried out as described in the manual. Instead of methyl blue, hematoxylin was used to counterstain the sections.

### Immunofluorescence staining

Paraffin sections were re-hydrated and heat-induced epitope retrieval was performed. NGS was used for blocking. Sections were stained with anti-K6 (905701, Biolegend), anti-K10 (905401, Biolegend) and anti-K14(MA5-11599, Invitrogen) overnight at 4 °C. Stainings were visualized using Alexa-488 (A11008/A11001, Molecular Probes) and Alexa-549 (A11012, Molecular probes) fluorescent-conjugated secondary antibodies and all sections were counterstained with DAPI.

Cells were seeded on chambers slides, stimulated over-night with Doxycycline (Sigma-Aldrich D9891) and fixed for 20 min with 4% PFA. Cells were washed 3x with PBS, blocked with 1% Triton X in PBS containing 1% BSA for 30 min and stained with a-ZBP1 antibody (Adipogen, clone Zippy-1, AG-20B-0010-C100) in 0.1% Triton X with 1% BSA for 1 h at RT. Cells were washed 3x with PBS followed by staining with Alexa-488 (A11008/A11001, Molecular Probes) fluorescent-conjugated secondary antibody and counterstaining with DAPI.

### Immunoblotting

Skin tissue samples were homogenized using Precellys tissue homogenizer in protein lysis buffer supplemented with protease and phosphatase inhibitors. Cells were lysed in protein lysis buffer supplemented with protease and phosphatase inhibitors for 20 min on ice. Cell suspension was centrifuged at 13,000 rpm and the protein concentration in the supernatant was measured using PIERCE 600 nm Protein Assay Reagent. Lysates were adjusted to 2 µg/µl. SDS Laemmli loading buffer (1 mM DTT) was added and samples were boiled at 95 °C for 8 min. Protein lysates were separated using SDS-Gel electrophoresis and transferred to PDVF-membranes (IPVH00010). Membranes were blocked for 1 h in 5% milk/0.1% PBS-T and incubated overnight at 4° in primary antibodies against a-Tubulin (T6074, Sigma), a-ZBP1 (Adipogen AG-20B-0010), a-RIPK3 (Enzo Life Sciences, ADI-095-242-100) and a-RIPK1(3492 Cell signaling technology). Membranes were washed 3x for 10 min in 0.1% PBS-T and probed with secondary HRP-coupled antibody (anti-mouse or anti-rabbit, GE Healthcare, Jackson ImmunoResearch) for 1 h. Proteins were detected using ECL Western Blotting Detection Reagent (RPN2106, GE Healthcare) and SuperSignal West Pico PLUS Chemiluminescent substrate (34580, Thermo Scientific).

### Immunoprecipitation

iMEFs were plated 24 h before stimulation in a 10 cm plate (3 × 10^6^ cells/well). Cells were stimulated with Doxycycline (Sigma-Aldrich D9891) for 12 h, washed 3x with ice-cold PBS and lysed for 20 min on ice using ALB lysis buffer (30 mM Tris HCl pH 7.5, 40 mM NaCl, 2 mM EDTA, 2 mM KCL, 10% Glycerin, 1% Triton x 100). The lysates were centrifuged for 30 min at 13.000 (4 °C) and protein concentration was calculated using PIERCE 600 nm Protein Assay Reagent. M2 Flag beads were washed 3x in lysis buffer, resuspend in an appropriate volume and 100 µl of beads (20 µl beads for 2 × 10^7^ cells) was added to protein lysates. Lysates were incubated moving ON, washed 3x with lysis buffer on the next day and boiled at 95 °C for 8 min in 50 µl Laemmli + 1 mM DTT. Samples were immunoblotted as described above.

### qRT-PCR

Total RNA was extracted from abdominal skin tissue using Trizol (Life Technologies) and RNeasy Columns (Qiagen) or the Direct-zol RNA isolation kit (Biozol). cDNA was prepared with the LUNA RT SuperMix Kit (E3010L, NEC). qRT-PCR was performed with TaqMan probes (Thermo Scientific, *Il1b* Mm00434228_m1, *Il6* Mm00446190_m1, *Tnf* Mm00443258_m1, *Ifi44* Mm00505670_m1, *Oasl1* Mm00455081_m1, *Ifit1* Mm00515153_m1, *Zbp1* Mm00457979_m1 *Ccl4* *Mm00443111_m1* *Cxcl9* Mm00434946_m1) in duplicates for each sample, using *Tbp* (Mm00446973_m1) as a reference gene. Relative expression of gene transcript was analyzed by 2^−^^ΔΔ CT^. In all graphs healthy littermate controls as well as the original *R26*^*LSL.ZBP2ca*^ controls were used. Outliers were excluded using the GraphPad Prism outlier function.

## Results

### MLKL-mediated necroptosis drives skin inflammation in FADD^E-KO^ mice

**Fig. 1:**
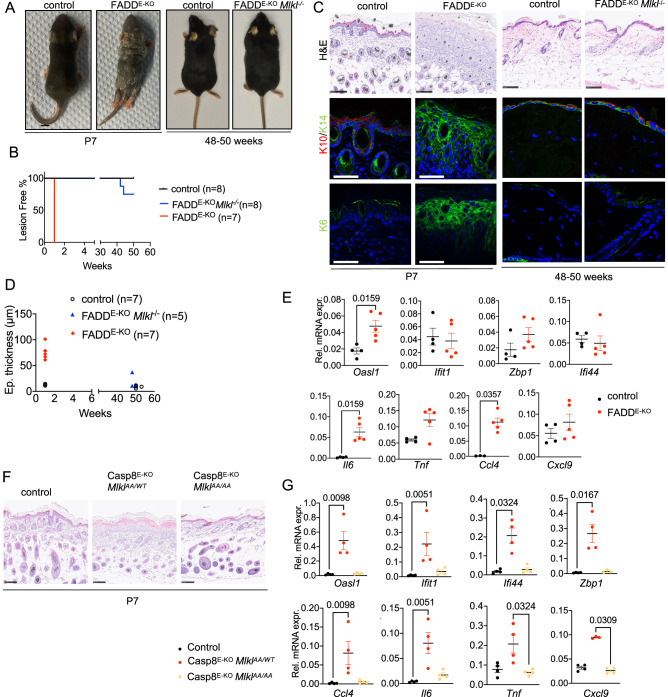
MLKL-mediated necroptosis causes skin inflammation in FADD^E-KO^ mice. **A** Representative photographs of FADD^E-KO^ (*n* = 7) mouse with unaffected littermate (*n* = 4) at P7 and of FADD^E-KO^ *Mlkl*^*−/−*^ (*n* = 8) mouse with WT littermate (*n* = 8) at 48–50 weeks. **B** Graph depicting lesion onset of mice with indicated genotypes. **C** Representative images from skin sections of FADD^E-KO^ mouse, FADD^E-KO^ *Mlkl*^*−/−*^ mouse and littermates stained with H&E (Scale bars = 100 µM, *n* = 7 for FADD^E-KO^, *n* = 4 for control, *n* = 5 for FADD^E-KO^ *Mlkl*^*−/−*^, *n* = 4 for control) or immunostained with K10, K14 and K6 (Scale bars = 50 µM, *n* = 4 for FADD^E-KO^, *n* = 3 for control, *n* = 5 for FADD^E-KO^ *Mlkl*^*−/−*^, *n* = 4 for control). **D** Graph depicting epidermal thickness of mice with indicated genotypes. **E** Graphs depicting relative mRNA expression of the indicated cytokines and ISGs in RNA from whole-skin tissue of mice at P7 of the indicated genotypes measured by qRT-PCR. Each dot represents one mouse. Mean ± SEM are shown. **F** Representative images from skin sections of Casp8^E-KO^ *Mlkl*^*AA/WT*^, Casp8^E-KO^ *Mlkl*^*AA/AA*^ and healthy control stained with H&E (Scale bar 100 µM, *n* = 4 for Casp8^E-KO^ *Mlkl*^*AA/WT*^, *n* = 4 for Casp8^E-KO^ *Mlkl*^*AA/AA*^, *n* = 4 for control). **G** Graphs depicting relative mRNA expression of the indicated cytokines and ISGs in RNA from whole-skin tissue of mice at P7-10 of the indicated genotypes measured by qRT-PCR). Statistical significance was determined using Kruskal–Wallis test (one-sided) in (**E**) and (**G**).

We showed previously that mice lacking FADD specifically in epidermal keratinocytes (*Fadd*^fl/fl^ *K14-Cre*, hereafter referred to as FADD^E-KO^ mice) developed severe inflammatory skin lesions that were prevented by RIPK3 deficiency, suggesting that necroptosis of FADD-deficient keratinocytes drives skin inflammation [21]. Since RIPK3 has been shown to induce also necroptosis-independent inflammatory responses [11, 25], to unequivocally prove that necroptosis drives the pathology in FADD^E-KO^ mice we crossed them with mice lacking MLKL. FADD^E-KO^ mice developed inflammatory skin lesions starting at postnatal day 6 (P6), which progressed rapidly to a severe dermatitis requiring their sacrifice by P7 (Fig. 1A, B). Histologically, the skin of FADD^E-KO^ mice at P7 was characterized by epidermal thickening, increased expression of the basal keratinocyte marker keratin 14 (K14), loss of the suprabasal keratinocyte marker keratin 10 (K10), as well as aberrant expression of keratin 6 (K6), which serves as a marker for an inflamed and hyperplastic epidermis (Fig. 1C, D). Moreover, FADD^E-KO^ mice at P7 displayed increased expression of the inflammatory cytokines *Tnf* and *Il6* as well as of the chemokines *Ccl4* and *Cxcl9* and certain interferon-stimulated genes (ISGs) including *Oasl1* and *Zbp1* (Fig. 1E). MLKL deficiency prevented the development of severe skin lesions in FADD^E-KO^ mice (Fig. 1A–C). When followed up to the age of 1 year, only 2 out of 8 FADD^E-KO^ *Mlkl*^−/−^ mice showed minor skin lesions at the age of 40–50 weeks (Fig. 1B). Mice lacking caspase 8 specifically in keratinocytes (*Casp8*^*fl/fl*^ *K14Cre*, hereafter referred to as Casp8^E-KO^) developed inflammatory skin lesions similarly to FADD^E-KO^ mice (Fig. 1F). To address whether MLKL-dependent necroptosis drives skin inflammation also in Casp8^E-KO^ mice, we crossed them to *Mlkl*^AA/AA^ mice, which express a mutated version of MLKL that cannot be phosphorylated by RIPK3 due to substitution of serines at positions 345 and 347 to alanines (Supplementary Fig. 1A, B) [26–28]. To confirm that these substitutions prevented activation of MLKL and necroptosis, we analyzed side by side primary BMDMs and lung fibroblasts from *Mlkl*^*AA/AA*^ and from *Mlkl*^-/-^ mice generated by CRISPR/Cas9-mediated targeting of the *Mlkl* gene (Supplementary Fig. 1C). Cells from *Mlkl*^*AA/AA*^ mice expressed normal levels of MLKL, but did not show MLKL phosphorylation and were fully protected from necroptosis induced by treatment with TNF together with the SMAC-mimetic compound birinapant and the caspase inhibitors Emricasan or Z-VAD, similarly to *Mlkl*^−/−^ BMDMs (Supplementary Fig. 1D, E), demonstrating that mutation of the RIPK3 phosphorylation sites fully prevented MLKL-mediated necroptosis. Casp8^E-KO^ *Mlkl*^AA/AA^ mice did not develop inflammatory skin lesions showing that mutation of the RIPK3 phosphorylation sites prevented necroptosis also in vivo (Fig. 1F). Importantly, the skin of Casp8^E-KO^ *Mlkl*^AA/AA^ mice did not show upregulation of inflammatory genes and ISGs, demonstrating that keratinocyte necroptosis drives the expression of these genes in Casp8^E-KO^ mice (Fig. 1G). Collectively, these results demonstrated that MLKL-mediated keratinocyte necroptosis causes inflammation in FADD^E-KO^ and Casp8^E-KO^ mice.

### ZBP1 promotes TNFR1-independent skin inflammation in FADD^E-KO^ mice

**Fig. 2:**
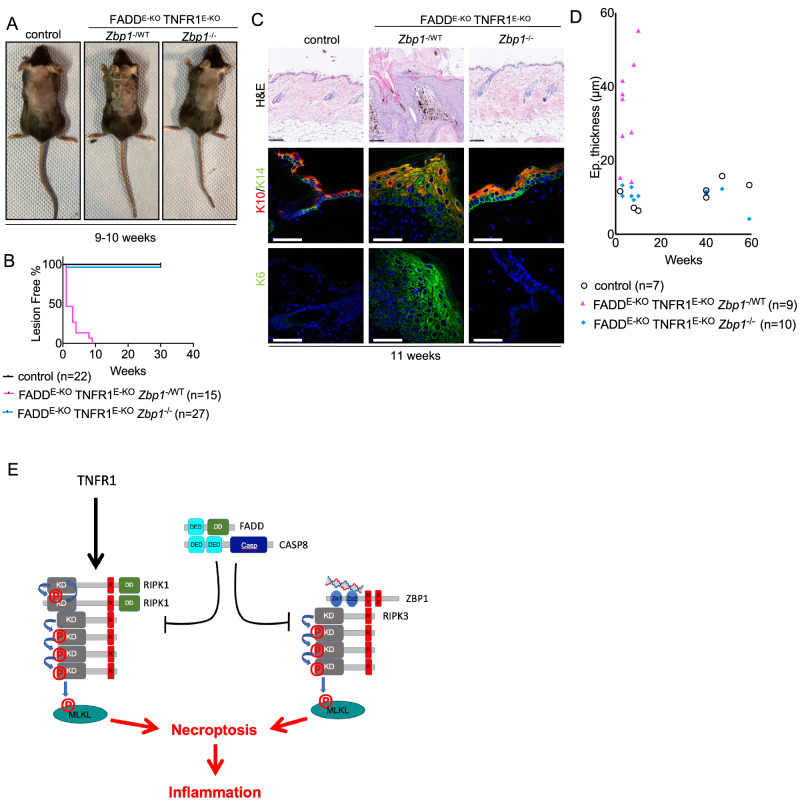
TNFR1 and ZBP1 synergize to drive skin inflammation in FADD^E-KO^ mice. **A** Representative photographs of FADD^E-KO^ TNFR1^E-KO^ *Zbp1*^−/WT^ (*n* = 15), FADD^E-KO^ TNFR1^E-KO^ *Zbp1*^−/−^ (*n* = 27)  and control  (*n* = 22) mice. **B** Graph depicting lesion onset of mice with indicated genotypes. **C** Representative images from skin sections stained with H&E (Scale bars = 100 µM, *n* = 9 for FADD^E-KO^ TNFR1^E-KO^ *Zbp1*^−*/WT*^, *n* = 10 for FADD^E-KO^ TNFR1^E-KO^ *Zbp1*^*−/−*^, *n* = 7 for control) or immunostained with K10, K14 and K6 (Scale bars = 50 µM, *n* = 3 for FADD^E-KO^ TNFR1^E-KO^ *Zbp1*^−*/WT*^ *n* = 5 for FADD^E-KO^ TNFR1^E-KO^ *Zbp1*^−/−^, *n* = 6 for control). **D** Graph depicting epidermal thickness of mice with indicated genotypes. **E** Model depicting pathology induced by loss of FADD and loss of FADD in combination with loss of TNFR1 in keratinocytes.

We showed previously that TNFR1 deficiency delayed but could not prevent the development of inflammatory skin lesions in FADD^E-KO^ mice, suggesting that additional, TNFR1-independent, mechanisms drive keratinocyte necroptosis and skin inflammation in these animals [21]. Our previous studies identified ZBP1 as a potent driver of keratinocyte necroptosis and skin inflammation in mice lacking RIPK1 in keratinocytes or expressing RIPK1 with mutated RHIM [6, 13], suggesting that ZBP1 could be implicated in inducing keratinocyte necroptosis in FADD^E-KO^ mice. Yet, ZBP1 deficiency could not ameliorate the skin pathology in FADD^E-KO^ mice [6], indicating that ZBP1 does not play an essential role in this model. However, our previous work in mice lacking FADD in intestinal epithelial cells (IECs) showed that TNFR1 cooperates with ZBP1 to induce necroptosis-mediated gut inflammation, revealing an intricate interplay between TNFR1 and ZBP1 in causing IEC necroptosis [11]. We therefore hypothesized that ZBP1 might drive TNFR1-independent skin inflammation in FADD^E-KO^ mice. To address this hypothesis, we generated and analyzed *Fadd*^*fl/fl*^ *Tnfr1*^*fl/fl*^ *K14-Cre Zbp1*^*−/−*^ (hereafter referred to as FADD^E-KO^ TNFR1^E-KO^ *Zbp1*^*−/−*^) mice, which lack FADD and TNFR1 in keratinocytes in a ZBP1-deficient genetic background. Consistent with our earlier findings that ubiquitous TNFR1 deficiency delayed but could not prevent skin inflammation in FADD^E-KO^ mice, *Fadd*^*fl/fl*^ *Tnfr1*^*fl/fl*^ *K14-Cre Zbp1*^*-/WT*^ (hereafter referred to as FADD^E-KO^ TNFR1^E-KO^ *Zbp1*^*−/WT*^) mice, which lack FADD and TNFR1 specifically in epidermal keratinocytes and are heterozygous for the *Zbp1* knockout allele, developed progressive skin lesions between 3–10 weeks of life that reached pre-determined termination criteria requiring the humane sacrifice of the animals by the age of 11 weeks (Fig. 2A, B). In contrast, we did not observe any skin lesions in FADD^E-KO^ TNFR1^E-KO^ *Zbp1*^*−/−*^ mice up to at least 30 weeks of age (Fig. 2A, B). Histopathological analysis revealed that combined loss of TNFR1 and ZBP1 fully prevented the development of inflammatory skin lesions in FADD^E-KO^ mice (Fig. 2C, D). Whereas skin sections from FADD^E-KO^ TNFR1^E-KO^ *Zbp1*^*−/WT*^ littermates showed the characteristic epidermal hyperplasia and altered keratin expression, the skin of FADD^E-KO^ TNFR1^E-KO^ *Zbp1*^*−/−*^ mice showed normal epidermal thickness and differentiation with no signs of skin pathology (Fig. 2C, D). Collectively, these results showed that ZBP1 and TNFR1 cooperate to induce keratinocyte necroptosis and skin inflammation in FADD^E-KO^ mice (Fig. 2E).

### C-terminally truncated ZBP1 induces cell death in the absence of caspase inhibitors

**Fig. 3:**
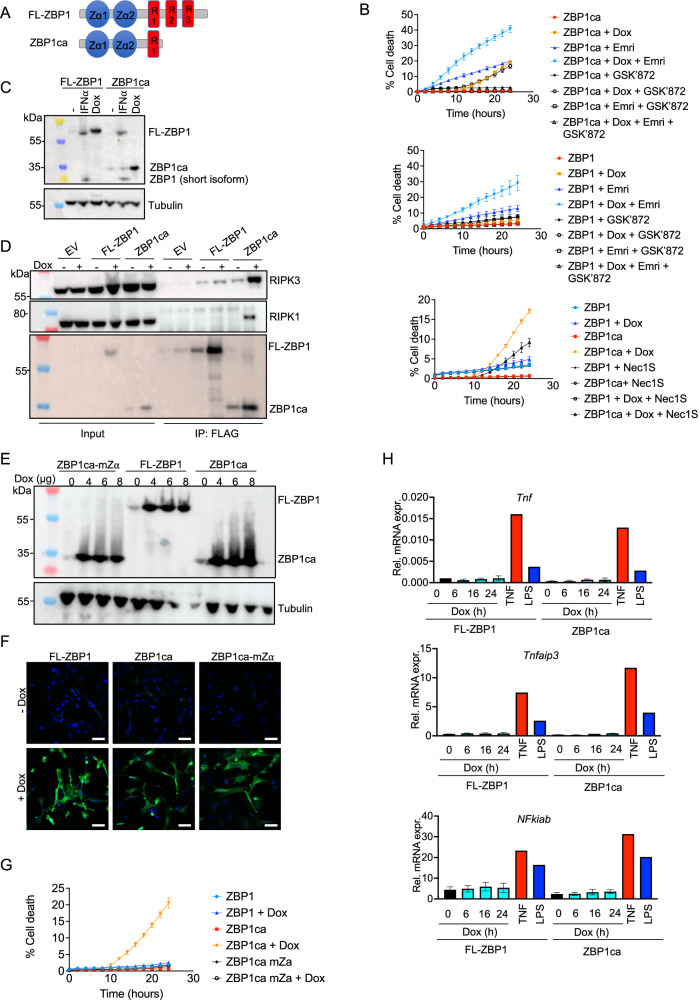
C-terminally truncated ZBP1 induces cell death in vitro. **A** Scheme depicting structure of FL-ZBP1 and ZBP1ca. **B** Cell death measured by Draq7 uptake in iMEFs stimulated with combinations of 1 µg doxycycline (dox), emricasan (emri), Nec1s and RIPK3 inhibitor GSK’827. Cell death was normalized to 2 h of Draq5 treatment for each genotype. Representative of at least 3 different experiments. Mean ± SEM are shown. **C** Immunoblot analysis of protein extracts from iMEFs transduced with doxycycline (Dox)-inducible FL-ZBP1- or ZBP1ca-expressing vectors or stimulated with IFNα. Representative of 3 different experiments. **D** Anti-Flag immunoprecipitates in iMEFs expressing dox-induced Flag-tagged FL-ZBP1 or Flag-tagged ZBP1ca stimulated with dox. Representative of 5 different experiments. **E** Immunoblot analysis of protein extracts from iMEFs transduced with Dox-inducible ZBP1ca-, FL-ZBP1 or ZBP1ca mZα-expressing vectors after treatment with increasing Dox concentrations. Representative of 3 different experiments. **F** Representative images of iMEFs expressing the indicated ZBP1 constructs after overnight doxycycline (+) treatment immunostained for ZBP1. **G** Cell death measured by Draq7 uptake in iMEFs stimulated with 8 µg dox. Representative of 3 different experiments. Mean ± SEM are shown. **H** Relative mRNA expression of RNA from iMEFs expressing FL-ZBP1 or ZBP1ca after indicated times of dox treatment. Graph shows mean ± SEM of three different experiments, the same LPS + TNF treated control RNA was used for all experiments.

Our results described above, together with our previous findings that ZBP1 causes MLKL-mediated skin inflammation in mice lacking RIPK1 or expressing RIPK1 with mutated RHIM, showed that ZBP1 is a potent driver of keratinocyte necroptosis [6, 13]. However, in these models the absence of FADD or RIPK1 sensitizes cells to ZBP1-induced necroptosis, therefore it remains unclear whether ZBP1 also causes necroptosis in cells with intact FADD-caspase-8 and RIPK1 signaling. To this end, we sought to develop an experimental system allowing to induce ZBP1-mediated death in cells with normal expression of other cell death regulators including FADD, caspase-8 and RIPK1. As shown previously [13], doxycycline-induced overexpression of wild-type full-length mouse ZBP1 (FL-ZBP1) could induce cell death in immortalized mouse embryonic fibroblasts (iMEFs) in the presence, but not in the absence, of the caspase inhibitor emricasan (also known as IDN-6556, Fig. 3A, B). In order to identify a form of ZBP1 that can induce cell death also in the absence of caspase inhibitors, we generated and tested different versions of C-terminally truncated ZBP1 proteins. We found that deletion of the last 203 amino acids resulting in the expression of a C-terminally truncated mouse ZBP1 containing the two Zα domains and the first RHIM was capable of inducing cell death in iMEFs in the absence of emricasan (Fig. 3A, B). Hereafter, we refer to this C-terminally truncated ZBP1 version as constitutively active ZBP1, abbreviated as “ZBP1ca”. ZBP1ca and FL-ZBP1 were expressed at similar levels after doxycycline induction (Fig. 3C), which were moderately increased compared to endogenous ZBP1 induced by treatment with IFNα (Fig. 3C).

ZBP1ca-mediated cell death could be enhanced by additional emricasan treatment but could not be blocked by the RIPK3 kinase inhibitor GSK’872 (Fig. 3B). Importantly, combined treatment with emricasan and GSK’872 fully prevented ZBP1ca-induced cell death (Fig. 3B), showing that ZBP1ca triggers RIPK3 kinase dependent necroptosis as well as caspase-8-mediated apoptosis. Interestingly, inhibition of RIPK1 kinase activity with Necrostatin-1s (Nec1s) could partially ameliorate ZBP1ca-induced cell death (Fig. 3B), suggesting that ZBP1ca could also interact with and activate RIPK1. This finding was unexpected, as in our previous studies we found that, in the absence of caspase inhibitors, ZBP1 could interact with RIPK3 but not with RIPK1 [6, 13]. In order to investigate whether the truncated ZBP1ca could interact with RIPK1, we immunoprecipitated FL-ZBP1 and ZBP1ca using anti-FLAG antibodies followed by immunoblotting for RIPK3 and RIPK1. Consistent with our earlier results [6, 13], we could detect RIPK3 but not RIPK1 after immunoprecipitation of overexpressed FL-ZBP1 (Fig. 3D). In contrast to the full-length protein, RIPK1 co-immunoprecipitated with ZBP1ca (Fig. 3D). Moreover, increased amounts of RIPK3 were detected in the ZBP1ca immunoprecipitates indicating that the truncated protein interacted more strongly with RIPK3 (Fig. 3D). This result, together with the finding that Nec1s treatment partially prevented ZBP1ca-induced cell death, suggested that ZBP1ca interacts with and activates RIPK1 to induce cell death. To assess whether ZBP1ca-mediated cell death requires sensing of a ligand via its Zα domains, we employed ZBP1ca with mutations of both Zα domains (hereafter referred to as ZBP1ca-mZα). Immunoblot and immunofluorescence analysis revealed similar expression levels of ZBP1ca-mZα compared to ZBP1ca in doxycycline treated cells (Fig. 3E, F). However, ZBP1ca-mZα expression did not trigger cell death in iMEFs, showing that ZBP1ca-mediated death depends on sensing of a ligand via its Zα domains (Fig. 3G). It has been reported previously that ZBP1 activates NF-κB signaling, suggesting that it could contribute to inflammation in a cell death-independent manner [4, 5, 29]. We therefore assessed whether doxycycline-inducible expression of ZBP1 or ZBP1ca could promote NF-κB-dependent gene expression. Whereas LPS or TNF treatment induced the expression of *Tnf*, *Tnfaip3* and *Nfkiab* mRNAs, we did not detect upregulation of these genes following ZBP1 or ZBP1ca expression. Therefore, neither ZBP1 nor ZBP1ca expression could induce the expression of NF-κB target genes (Fig. 3H). Collectively, these results showed that ZBP1ca triggered cell death by inducing RIPK3-MLKL-dependent necroptosis and caspase-8 dependent apoptosis.

### ZBP1ca expression in keratinocytes causes skin inflammation

**Fig. 4:**
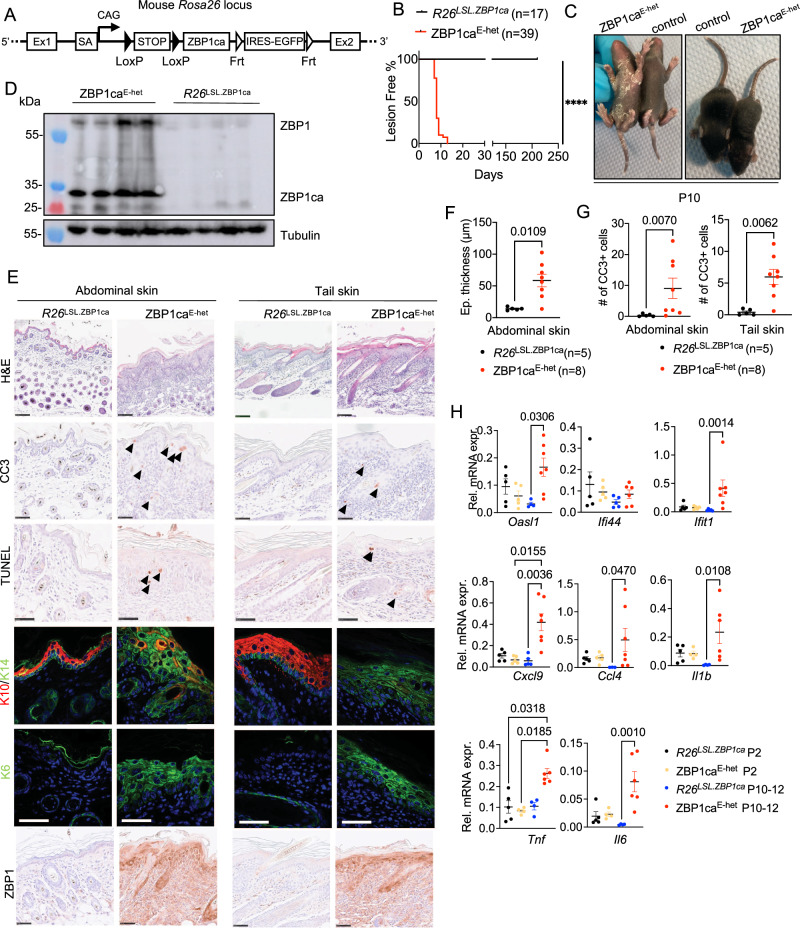
ZBP1ca expression in keratinocytes causes skin inflammation. **A** Targeting scheme for generation of *R26*^*LSL.ZBP1ca*^ mouse strain using CRISPR Cas9. **B** Graph depicting lesion onset of mice with indicated genotypes. **C** Representative photographs of ZBP1ca^E-het^ mouse (*n* = 39) and WT littermate (*n* = 17) at P10. **D** Immunoblot analysis of whole skin protein lysates from ZBP1ca^E-het^ (*n* = 4) and littermate controls (*n* = 4). Mice were analyzed at P10–12. **E** Representative images from abdominal and tail skin sections of ZBP1ca^E-het^ mice and littermates at P10–12 stained with H&E (Scale bars = 100 µM, *n* = 11 for ZBP1ca^E-het^, *n* = 5 for control), CC3^+^ (*n* = 5 for ZBP1ca^E-het^, *n* = 2 for *R26*^*LSL.ZBP1ca*^*)*, TUNEL (*n* = 4 for ZBP1ca^E-het^, *n* = 2 for *R26*^*LSL.ZBP1ca*^*)*, or immunostained for ZBP1 (Scale bars = 50 µM, *n* = 6 for ZBP1ca^E-het^, *n* = 2 for control) or K10, K14 and K6 (Scale bars = 50 µM, *n* = 9 for ZBP1ca^E-het^, *n* = 11 for control). **F** Graph depicting epidermal thickness of mice with indicated genotypes at P10–12. Mean + SEM are shown. Each dot represents one mouse. **G** Graph showing the amount of CC3^+^ cells in abdominal and tail skin of mice with indicated genotypes. Mean ± SEM are shown. Each dot represents one mouse. **H** Graphs depicting relative mRNA expression of the indicated cytokines and ISGs in RNA from whole-skin tissue of P2 and of P10–12 mice of the indicated genotypes measured by qRT-PCR. Each dot represents one mouse. Mean ± SEM are shown. Statistical significance was determined using Kruskal–Wallis test (one-sided) in (**F**–**H**).

Our results showing that ZBP1ca expression caused cell death in iMEFs prompted us to employ ZBP1ca expression as a tool to study ZBP1-mediated signaling in vivo. To this end, we generated knock-in mice that express ZBP1ca under the control of the ubiquitously expressed *Rosa26* locus after Cre-mediated excision of a loxP-flanked stop cassette (*R26*^*LSL.ZBP1ca*^) (Fig. 4A). To study the effect of ZBP1ca expression in epidermal keratinocytes, we crossed *R26*^*LSL.ZBP1ca*^ animals with *K14-Cre* transgenics [20] to generate *R26*^*LSL.ZBP1ca/WT*^ *K14-Cre* mice (hereafter referred to as ZBP1ca^E-het^). ZBP1ca^E-het^ mice were born at the expected Mendelian ratio and were macroscopically indistinguishable from their *R26*^*LSL.ZBP1ca/WT*^ littermates until P7, when they started to develop skin lesions mainly affecting the abdominal and tail skin. The phenotype progressed rapidly to severe lesions, characterized by skin thickening and scaling gradually increasing in size and distribution (Fig. 4B, C). All ZBP1ca^E-het^ animals had to be euthanized between P9 and P14 because of reaching pre-determined ethical endpoints especially due to severe inflammation affecting the entire tail.

In order to characterize the skin pathology, we examined ZBP1ca^E-het^ mice at different time points after birth. At P2, ZBP1ca^E-het^ mice were macroscopically indistinguishable from their *R26*^*LSL.ZBP1ca/WT*^ littermates (Supplementary Fig. 2A). Immunohistological analysis of sections from abdominal and tail skin revealed expression of ZBP1ca in keratinocytes and an overall normal epidermal thickness and differentiation as judged by expression of keratin 14 (K14) in basal and K10 in suprabasal keratinocytes (Supplementary Fig. 2B). However, ZBP1ca^E-het^ mice showed upregulation of K6, a marker of skin inflammation, in interfollicular epidermis in both the abdominal and tail skin indicating the initiation of an inflammatory skin reaction already at P2 (Supplementary Fig. 2B). Histopathological analysis of abdominal and tail skin sections of mice sacrificed at P10–12 revealed pronounced epidermal thickening in ZBP1ca^E-het^ mice compared to their littermate controls (Fig. 4E, F). Immunoblot analysis of whole skin protein lysates from ZBP1ca^E-het^ mice revealed strong expression of ZBP1ca as well as a mild upregulation of endogenous ZBP1 (Fig. 4D). Immunohistochemical staining for ZBP1 confirmed expression of ZBP1ca in the epidermis but also revealed staining in the dermis, indicating the upregulation of endogenous ZBP1 in stromal and immune cells (Fig. 4E). The epidermis of ZBP1ca^E-het^ mice showed a nearly complete loss of K10 concomitant with a strong upregulation of K14 and K6 in all epidermal layers, consistent with strong epidermal hyperplasia and loss of differentiation (Fig. 4E). To assess whether ZBP1ca expression induced keratinocyte death, we performed immunostaining for cleaved caspase-3 (CC3) and TUNEL assays that revealed increased numbers of dying keratinocytes in the skin of ZBP1ca^E-het^ mice (Fig. 4E, G). To assess inflammatory gene expression, we performed qRT-PCR analysis on RNA isolated from abdominal skin of ZBP1ca^E-het^ and littermate control mice at P2 and at P10–12. These experiments revealed upregulation of inflammatory cytokines and chemokines such as *Tnf*, *Il-1β*, *Il6*, *Cxcl9* and *Ccl4*, as well as of ISGs including *Oasl1*, *Ifi44*, and *Ifit1* in the skin of ZBP1ca^E-het^ mice at the age of P10–12 compared to *R26*^*LSL.ZBP1ca/WT*^ controls (Fig. 4H). Importantly, these genes were not upregulated in ZBP1ca^E-het^ mice at P2, consistent with the absence of skin lesions at this stage. Taken together, these results showed that expression of ZBP1ca induced keratinocyte death and skin inflammation in vivo.

### RHIM-dependent RIPK3 signaling contributes to skin inflammation in ZBP1ca^E-het^ mice

**Fig. 5:**
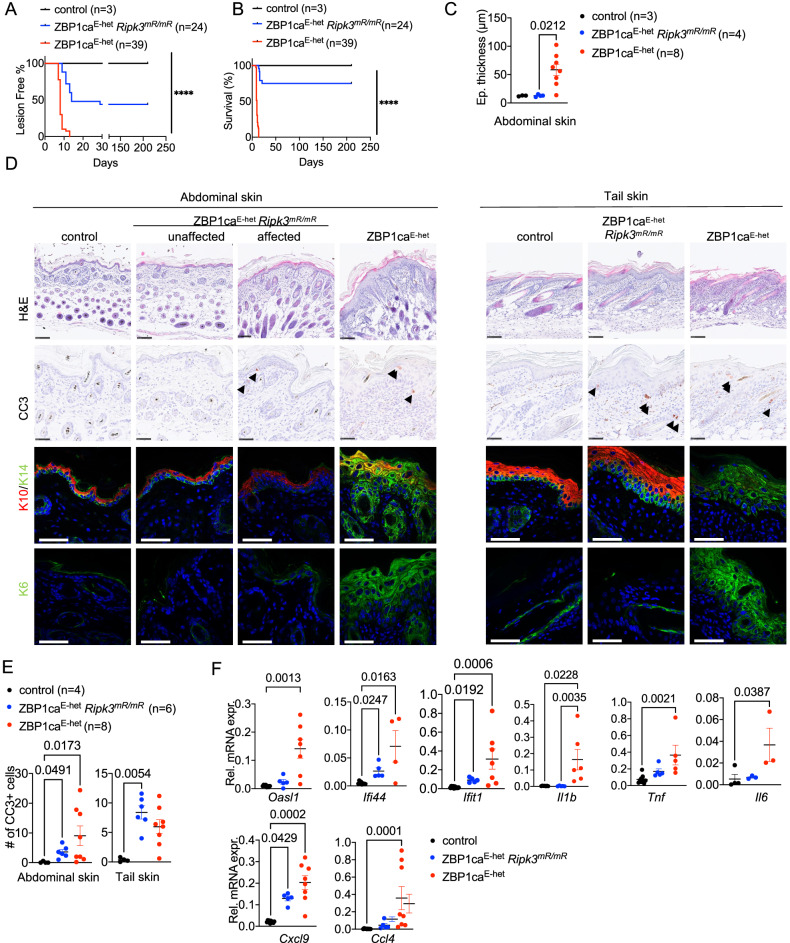
RIPK3 RHIM-dependent signaling promotes skin inflammation in ZBP1ca^E-het^ mice. **A** Graph depicting lesion-free survival of ZBP1ca^E-het^, ZBP1ca^E-het^ *Ripk3*^*mR/mR*^ and healthy littermates. **B** Graph depicting survival curve of ZBP1ca^E-het^, ZBP1ca^E-het^ *Ripk3*^*mR/mR*^ and healthy littermates. **C** Graph depicting epidermal thickness of mice with indicated genotypes at P10–12. Mean + SEM are shown. One dot represents one mouse. Data from the same ZBP1ca^E-het^ mice are included in all Figures for comparison. **D** Representative images from abdominal and tail skin sections of ZBP1ca^E-het^ *Ripk3*^*mR/mR*^, ZBP1ca^E-het^ and control mice stained with H&E (Scale bars = 100 µM, *n* = 5 for ZBP1ca^E-het^ *Ripk3*^*mR/mR*^, *n* = 11 for ZBP1ca^E-het^, *n* = 3 for control), CC3^+^ (*n* = 5 for ZBP1ca^E-het^, *n* = 5 for ZBP1ca^E-het^ *Ripk3*^*mR/mR*^, *n* = 3 for control) or immunostained with K10, K14 and K6 (Scale bars = 50 µM, *n* = 5 for ZBP1ca^E-het^ *Ripk3*^*mR/mR*^, *n* = 11 for ZBP1ca^E-het^, *n* = 3 for control) at P10–12. **E** Graph showing the amount of CC3^+^ cells in belly and tail skin of mice with indicated genotypes. Mean ± SEM are shown. Each dot represents one mouse. Data from the same ZBP1ca^E-het^ mice are included in all Figures for comparison. **F** Graphs depicting relative mRNA expression of the indicated cytokines and ISGs in RNA from whole-skin tissue of P10–12 mice of the indicated genotypes measured by qRT-PCR. Each dot represents one mouse. Mean ± SEM are shown. Data from the same ZBP1ca^E-het^ mice are included in all Figures for comparison. Statistical significance was determined using Kruskal–Wallis test (one-sided) in (**C**, **E**, **F**).

ZBP1 was previously shown to mediate both apoptosis and necroptosis by interacting with and activating RIPK3 [6, 11, 13, 30, 31]. We therefore hypothesized, that ZBP1ca induces keratinocyte death and skin inflammation by interacting with RIPK3 via its RHIM. In order to study the role of RHIM-dependent RIPK3 signaling we generated knock-in mice expressing RIPK3 with mutated RHIM by substituting the four conserved amino acids (VQIG) at position 448-451 with alanines (hereafter referred to as *Ripk3*^*mR/mR*^) (Supplementary Fig. 3A, B). Primary lung fibroblasts (LFs) from *Ripk3*^*mR/mR*^ mice expressed normal levels of RIPK3 protein and were fully protected from necroptosis induced by treatment with TNF together with the SMAC mimetic birinapant and emricasan (TSE), confirming that the introduced mutation disrupted RIPK3-dependent signaling (Supplementary Fig. 3C, D). To assess the role of RHIM-dependent RIPK3 signaling in ZBP1ca-mediated cell death and inflammation, we generated and analyzed *R26*^*LSL.ZBP1ca/WT*^ *x K14-Cre x Ripk3*^*mR/mR*^ (ZBP1ca^E-het^ *Ripk3*^*mR/mR*^) mice. Surprisingly, RIPK3 RHIM mutation strongly ameliorated but could not fully prevent skin lesion development in ZBP1ca^E-het^ mice. Out of 24 animals observed, 14 animals developed inflammatory skin lesions, affecting mainly the tail skin (Fig. 5A). The lesions particularly in tail skin reached the level of severity requiring the humane sacrifice of 6 mice before the age of 4 weeks (Fig. 5B). In the remaining 18 mice, the lesions appeared to be transient and disappeared after a few weeks, with these animals remaining healthy at least up to the age of 30 weeks (Fig. 5B and Supplementary Fig. 4). Consistent with the macroscopic appearance, histopathological analysis revealed an overall milder phenotype in the affected ZBP1ca^E-het^ *Ripk3*^*mR/mR*^ mice compared to ZBP1ca^E-het^ animals at the age of 10 days. Most skin areas showed normal epidermal thickness and differentiation, with only small affected areas showing upregulation of K6 (Fig. 5C, D). Immunostaining for cleaved caspase-3 revealed the presence of apoptotic cells in the skin of ZBP1ca^E-het^ *Ripk3*^*mR/mR*^ mice. Interestingly, whereas the number of CC3^+^ cells was decreased in the abdominal skin of ZBP1ca^E-het^ *Ripk3*^*mR/mR*^ mice, we found similar if not slightly increased numbers of apoptotic cells in tail skin from ZBP1ca^E-het^ *Ripk3*^*mR/mR*^ compared to ZBP1ca^E-het^ mice (Fig. 5D, E). Consistent with the histological picture, mutation of the RIPK3 RHIM suppressed the expression of inflammatory cytokines and chemokines as well as of ISGs in the skin of ZBP1ca^E-het^ *Ripk3*^*mR/mR*^ compared to ZBP1ca^E-het^ mice (Fig. 5F). However, ZBP1ca^E-het^ *Ripk3*^*mR/mR*^ mice showed moderately elevated expression of *Cxcl9, Ifi44* and *Ifit1* and a trend toward increased expression of *Tnf* in their skin compared to *K14-Cre* negative control mice, further supporting that blocking RHIM-dependent RIPK3 signaling did not fully prevent skin inflammation. Therefore, mutation of the RIPK3 RHIM strongly ameliorated but could not fully prevent keratinocyte death and skin inflammation in ZBP1ca^E-het^ *Ripk3*^*mR/mR*^ mice, showing that ZBP1ca drives cell death and inflammation by both RIPK3-dependent and RIPK3-independent mechanisms.

### Combined ablation of caspase-8 and MLKL fully prevents ZBP1ca-driven skin lesion development

**Fig. 6:**
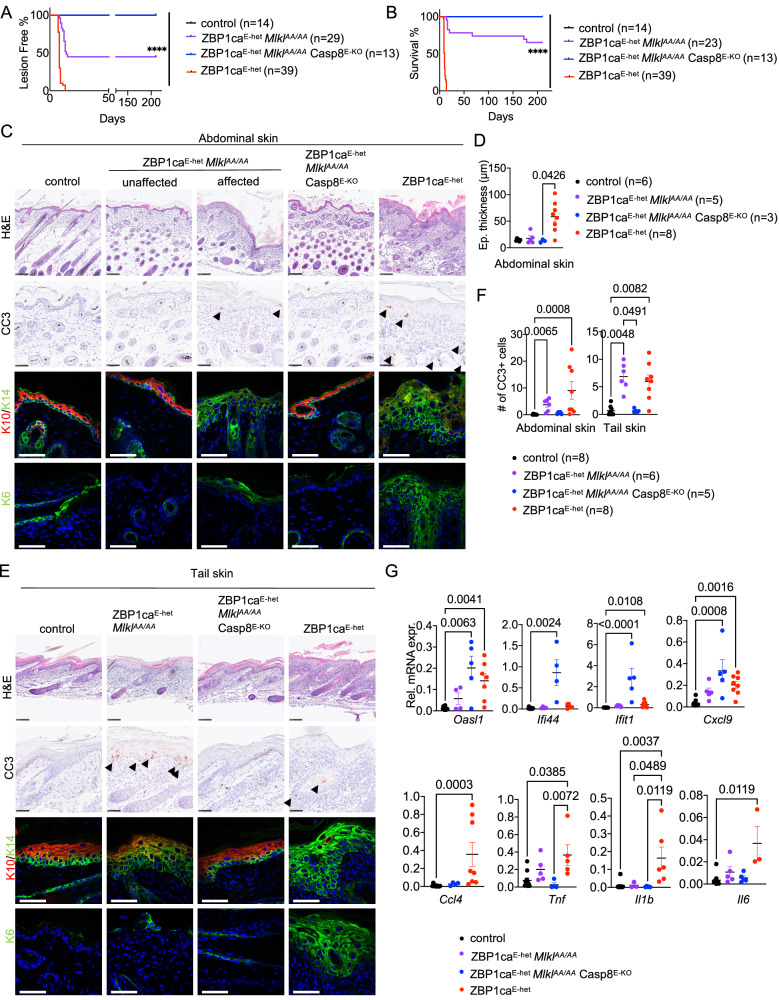
MLKL-dependent necroptosis and to a lesser extent caspase-8-dependent apoptosis cause skin inflammation in ZBP1ca^E-het^ mice. **A** Graph depicting lesion-free survival of ZBP1ca^E-het^, ZBP1ca^E-het^ *Mlkl*^*AA/AA*^, ZBP1ca^E-het^ *Mlkl*^*AA/AA*^ Casp8^E-KO^ and control mice. **B** Graph depicting survival curve of ZBP1ca^E-het^, ZBP1ca^E-het^ *Mlkl*^*AA/AA*^ and ZBP1ca^E-het^ *Mlkl*^*AA/AA*^ Casp8^E-KO^ mice with non-affected littermates. **C** Representative images from abdominal skin sections of ZBP1ca^E-het^, ZBP1ca^E-het^ *Mlkl*^*AA/AA*^ and ZBP1ca^E-het^ *Mlkl*^*AA/AA*^ Casp8^E-KO^ mice and control mice stained with H&E (Scale bars = 100 µM, *n* = 5 for ZBP1ca^E-het^ *Mlkl*^*AA/AA*^, *n* = 5 for ZBP1ca^E-het^ *Mlkl*^*AA/AA*^Casp8^E-KO^, *n* = 11 for ZBP1ca^E-het^, *n* = 8 for control), CC3^+^ (*n* = 5 for ZBP1ca^E-het^, *n* = 4 for ZBP1ca^E-het^ *Mlkl*^*AA/AA*^, *n* = 5 for ZBP1ca^E-het^ *Mlkl*^*AA/AA/*^ Casp8^E-KO^, *n* = 5 for control) or immunostained with K10, K14 and K6 (Scale bars = 50 µM, *n* = 4 for ZBP1ca^E-het^ *Mlkl*^*AA/AA*^, *n* = 5 for ZBP1ca^E-het^ *Mlkl*^*AA/AA*^ Casp8^E-KO^, *n* = 11 for ZBP1ca^E-het^, *n* = 5 for control) at P10–12. **D** Graph depicting epidermal thickness of mice with indicated genotypes at P10–12. Mean + SEM are shown. Each dot represents one mouse. Data from the same ZBP1ca^E-het^ mice are included in all Figures for comparison. **E** Representative images from tail skin sections of ZBP1ca^E-het^, ZBP1ca^E-het^ *Mlkl*^*AA/AA*^ and ZBP1ca^E-het^ *Mlkl*^*AA/AA*^ Casp8^E-KO^ mice and control mice stained with H&E (Scale bars = 100 µM, *n* = 5 for ZBP1ca^E-het^ *Mlkl*^*AA/AA*^, *n* = 5 for ZBP1ca^E-het^ *Mlkl*^*AA/AA*^ Casp8^E-KO^, *n* = 11 for ZBP1ca^E-het^, *n* = 8 for control), CC3^+^ (*n* = 5 for ZBP1ca^E-het^, *n* = 4 for ZBP1ca^E-het^ *Mlkl*^*AA/AA*^, *n* = 5 for ZBP1ca^E-het^ *Mlkl*^*AA/AA*^ Casp8^E-KO^, *n* = 5 for control) or immunostained with K10, K14 and K6 (Scale bars = 50 µM, *n* = 4 for ZBP1ca^E-het^ *Mlkl*^*AA/AA*^, *n* = 5 for ZBP1ca^E-het^ *Mlkl*^*AA/AA*^ Casp8^E-KO^, *n* = 11 for ZBP1ca^E-het^, *n* = 5 for control) at P10–12. **F** Graph showing the amount of CC3^+^ cells in abdominal and tail skin of mice with indicated genotypes. Mean ± SEM are shown. Each dot represents one mouse. Data from the same ZBP1ca^E-het^ mice are included in all Figures for comparison. **G** Graphs depicting relative mRNA expression of the indicated ISGs and cytokines in RNA from whole-skin tissue of P10–12 mice of the indicated genotypes measured by qRT-PCR. Each dot represents one mouse. Mean ± SEM are shown. Data from the same ZBP1ca^E-het^ and ZBP1ca^E-het^ *Mlkl*^*AA/AA*^ mice are included in all Figures for comparison. Statistical significance was determined using Kruskal–Wallis test (one-sided) in (**D**, **F**, **G**).

Given the surprising finding that mutation of the RIPK3 RHIM did not fully prevent skin lesion development in ZBP1ca^E-het^ mice, we set out to dissect the contribution of caspase-8-dependent apoptosis and MLKL-driven necroptosis in this setting. To assess the role of MLKL-induced necroptosis in the pathogenesis of the skin lesions in ZBP1ca^E-het^ mice, we crossed them with *Mlkl*^*AA/AA*^ mice to generate ZBP1ca^E-het^ *Mlkl*^*AA/AA*^ animals. Macroscopic assessment of ZBP1ca^E-het^ *Mlkl*^*AA/AA*^ mice revealed that inhibition of necroptosis strongly ameliorated but could not prevent skin lesion development. Specifically, 16 out of 29 ZBP1ca^E-het^ *Mlkl*^*AA/AA*^ mice observed developed inflammatory skin lesions, with the rest of the mice remaining lesion-free at least up to the age of 30 weeks (Fig. 6A and Supplementary Fig. 5). In 8 out of the 16 affected mice, the skin lesions progressed in severity particularly in the tail reaching the pre-determined termination criteria requiring euthanasia of the animals (Fig. 6B). In line with the macroscopic assessment, histopathological analysis revealed only focal areas displaying epidermal thickening and upregulation of K14 and K6 in abdominal skin sections from ZBP1ca^E-het^ *Mlkl*^*AA/AA*^ mice, with most skin remaining unaffected (Fig. 6C, D). However, the affected ZBP1ca^E-het^ *Mlkl*^*AA/AA*^ mice developed more pronounced skin lesions in the tail, characterized by hyperplasia and upregulation of K14 expression (Fig. 6E). Interestingly, the amount of CC3^+^ cells was decreased in the abdominal skin but increased in tail skin of ZBP1ca^E-het^ *Mlkl*^*AA/AA*^ compared to ZBP1ca^E-het^ mice (Fig. 6C, E, F), a trend also visible in ZBP1ca^E-het^ *Ripk3*^*mR/mR*^ mice (Fig. 5D, E), indicating that in the absence of MLKL-dependent necroptosis more cells shift to apoptosis. Furthermore, the expression of inflammatory cytokines and chemokines and ISGs was strongly suppressed in the skin of ZBP1ca^E-het^ *Mlkl*^*AA/AA*^ compared to ZBP1ca^E-het^ mice, however, ZBP1ca^E-het^ *Mlkl*^*AA/AA*^ animals showed a trend toward higher expression of *Oasl1*, *Cxcl9*, *Tnf* and *Il-6* compared to K14-Cre negative control mice (Fig. 6G). Collectively, these results showed that inhibition of MLKL-mediated necroptosis strongly ameliorated but could not fully prevent skin lesion development in ZBP1ca^E-het^ *Mlkl*^*AA/AA*^ mice, similarly to the inhibition of RHIM-dependent RIPK3 signaling, arguing that necroptosis-independent mechanisms also contribute to the pathology.

We reasoned that caspase-8-mediated cell death could mediate the necroptosis-independent pathology in ZBP1ca^E-het^ mice. However, because inhibition of FADD/caspase-8 signaling in keratinocytes triggers TNFR1- and ZBP1-mediated necroptosis and skin inflammation (Figs. 1 and 2), it is not possible to use conditional ablation of FADD or caspase-8 alone to assess the role of FADD/Caspase-8-induced signaling in ZBP1ca^E-het^ mice. For this reason, we generated and analyzed *R26*^*LSL.ZBP1ca/WT*^ *K14-Cre Mlkl*^*AA/AA*^ *Casp8*^FL/FL^ mice (hereafter referred to as ZBP1ca^E-het^ *Mlkl*^*AA/AA*^ Casp8^E-KO^), which express ZBP1ca and lack caspase-8 specifically in keratinocytes in the *Mlkl*^*AA/AA*^ background. Macroscopic and histological analysis revealed that ZBP1ca^E-het^ *Mlkl*^*AA/AA*^ Casp8^E-KO^ mice had a normal skin and did not develop any signs of pathology at least until the age of 30 weeks (Fig. 6A–F and Supplementary Fig. 5), demonstrating that combined ablation of caspase-8-mediated apoptosis and MLKL-dependent necroptosis fully prevented skin lesion development induced by ZBP1ca expression in keratinocytes. qRT-PCR analysis showed that the expression of inflammatory cytokines and chemokines was suppressed in the skin of ZBP1ca^E-het^ *Mlkl*^*AA/AA*^ Casp8^E-KO^ mice, consistent with the absence of inflammatory lesions (Fig. 6G). However, the expression of *Oasl1*, *Ifi44, Ifit1* and *Cxcl9* was considerably increased in ZBP1ca^E-het^ *Mlkl*^*AA/AA*^ Casp8^E-KO^ compared to ZBP1ca^E-het^ mice. Since Casp8^E-KO^ *Mlkl*^AA/AA^ mice did not show increased expression of these ISGs (Fig. 1G), these results suggest that caspase-8 deficiency in keratinocytes caused upregulation of ZBP1ca-mediated ISG expression. Collectively, these results showed that ZBP1ca expression caused skin inflammation by inducing RIPK3-MLKL-dependent necroptosis and to a lesser extent caspase-8-dependent apoptosis in keratinocytes.

### RHIM-dependent RIPK1 signaling causes necroptosis-independent skin inflammation in ZBP1ca^E-het^ mice

**Fig. 7:**
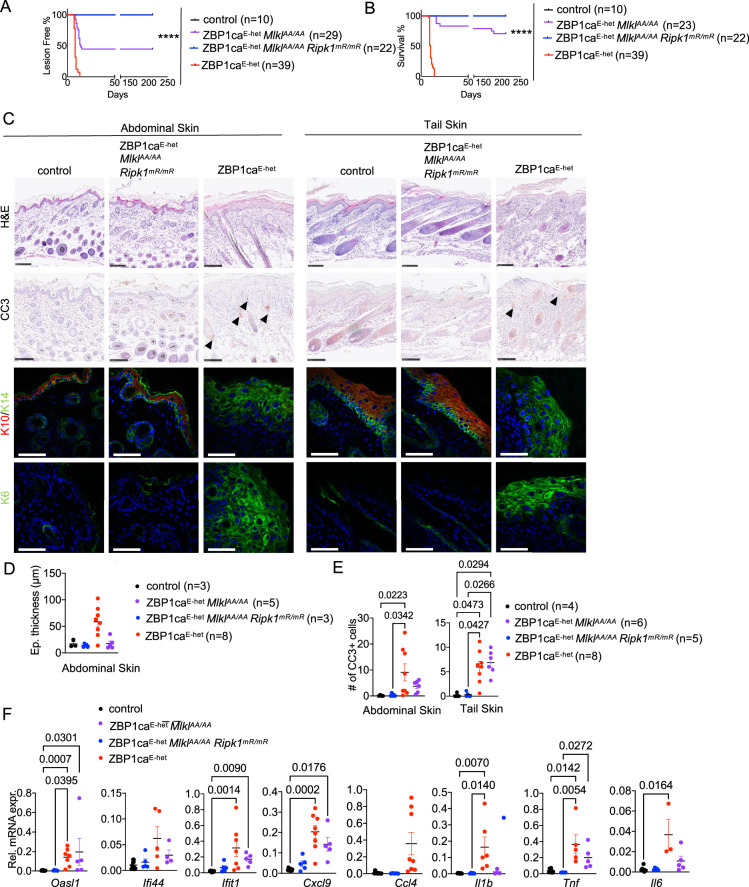
RIPK1 RHIM-dependent signaling causes MLKL-independent skin inflammation in ZBP1ca^E-het^ mice. **A** Graph depicting lesion-free survival of ZBP1ca^E-het^, ZBP1ca^E-het^ *Mlkl*^*AA/AA*^ and ZBP1ca^E-het^ *Mlkl*^*AA/AA*^ *Ripk1*^*mR/mR*^ mice and non-affected littermates. **B** Graph depicting survival curve of ZBP1ca^E-het^, ZBP1ca^E-het^ *Mlkl*^*AA/AA*^ and ZBP1ca^E-het^ *Mlkl*^*AA/AA*^ *Ripk1*^*mR/mR*^ mice with littermate controls. **C** Representative images from skin sections of ZBP1ca^E-het^ and ZBP1ca^E-het^ *Mlkl*^*AA/AA*^ *Ripk1*^*mR/mR*^ mice and control mice stained with H&E (Scale bars = 100 µM, *n* = 5 for ZBP1ca^E-het^ *Mlkl*^*AA/AA*^ *Ripk1*^*mR/mR*^, *n* = 11 for ZBP1ca^E-het^, *n* = 5 for control), CC3^+^ (Scale bars = 100 µM, *n* = 5 for ZBP1ca^E-het^ *Mlkl*^*AA/AA*^ *Ripk1*^*mR/mR*^, *n* = 5 for ZBP1ca^E-het^, *n* = 5 for control), or immunostained with K10, K14 and K6 (Scale bars = 50 µM, *n* = 5 for ZBP1ca^E-het^ *Mlkl*^*AA/AA*^ *Ripk1*^*mR/mR*^, *n* = 8 for ZBP1ca^E-het^, *n* = 5 for control) at P10–12. **D** Graph depicting epidermal thickness of mice with indicated genotypes at P10–12. Mean ± SEM are shown. Each dot represents one mouse. Data from the same ZBP1ca^E-het^ and ZBP1ca^E-het^ *Mlkl*^*AA/AA*^ mice are included in all Figures for comparison. **E** Graph showing the amount of CC3^+^ cells in abdominal and tail skin of mice with indicated genotypes. Mean ± SEM are shown. Each dot represents on mouse. Data from the same ZBP1ca^E-het^ and ZBP1ca^E-het^ *Mlkl*^*AA/AA*^ mice are included in all Figures for comparison. **F** Graphs depicting relative mRNA expression of the indicated cytokines in RNA from whole-skin tissue of P10–12 mice of the indicated genotypes measured by qRT-PCR. Each dot represents one mouse. Mean ± SEM are shown. Data from the same ZBP1ca^E-het^ and ZBP1ca^E-het^ *Mlkl*^*AA/AA*^ mice are included in all Figures for comparison. Statistical significance was determined using Kruskal–Wallis test (one-sided) in (**D**–**F**).

Our findings that inhibition of RHIM-dependent RIPK3 signaling did not fully prevent skin inflammation in ZBP1ca^E-het^ mice suggested that ZBP1ca could induce keratinocyte cell death by activating RIPK1. Moreover, whereas full length ZBP1 did not interact with RIPK1 unless caspases were inhibited [6, 13], ZBP1ca efficiently interacted with RIPK1 (Fig. 3D), further supporting that ZBP1ca could trigger RIPK1-mediated cell death and inflammation in the skin. Therefore, to address the role of RIPK1 we employed *Ripk1*^*mR/mR*^ mice, which express RIPK1 with mutated RHIM [6]. Because *Ripk1*^*mR/mR*^ mice die perinatally due to ZBP1-mediated RIPK3-MLKL-dependent necroptosis [6], it is not possible to study the role of RHIM-dependent RIPK1 signaling in ZBP1ca^E-het^ mice unless if necroptosis is inhibited. We therefore generated and analyzed *R26*^*LSL.ZBP1ca*^ *x K14-Cre x Mlkl*^*AA/AA*^ *x Ripk1*^*mR/mR*^ mice (hereafter referred to as ZBP1ca^E-het^ *Mlkl*^*AA/AA*^ *Ripk1*^*mR/mR*^), which express RIPK1 with mutated RHIM in the *Mlkl*^*AA/AA*^ background. ZBP1ca^E-het^ *Mlkl*^*AA/AA*^ *Ripk1*^*mR/mR*^ mice did not develop macroscopically visible skin lesions up to at least 30 weeks of age (Fig. 7A, B). Moreover, histological analysis of abdominal and tail skin from mice at P10 showed a healthy skin with normal epidermal thickness and differentiation without signs of inflammation and cell death (Fig. 7C–E). Additionally, qRT-PCR analysis of inflammatory cytokines and chemokines showed that the RIPK1 RHIM mutation also fully suppressed the upregulation of inflammatory cytokines and chemokines as well as ISGs in the skin of ZBP1ca^E-het^ *Mlkl*^*AA/AA*^ *Ripk1*^*mR/mR*^ mice. Therefore, inhibition of RHIM-dependent RIPK1 signaling prevented MLKL-independent skin inflammation induced by ZBP1ca expression in keratinocytes. Together with our findings that combined inhibition of MLKL-dependent necroptosis and caspase-8-dependent apoptosis fully prevented skin lesion development in ZBP1ca^E-het^ mice, these results showed that ZBP1ca caused skin inflammation by inducing RIPK3-MLKL-dependent necroptosis and to a lesser extent RIPK1-mediated caspase-8-dependent cell death in keratinocytes.

### RIPK1 mediates apoptosis and skin inflammation in ZBP1ca^E-het^ mice independently of its kinase activity

**Fig. 8:**
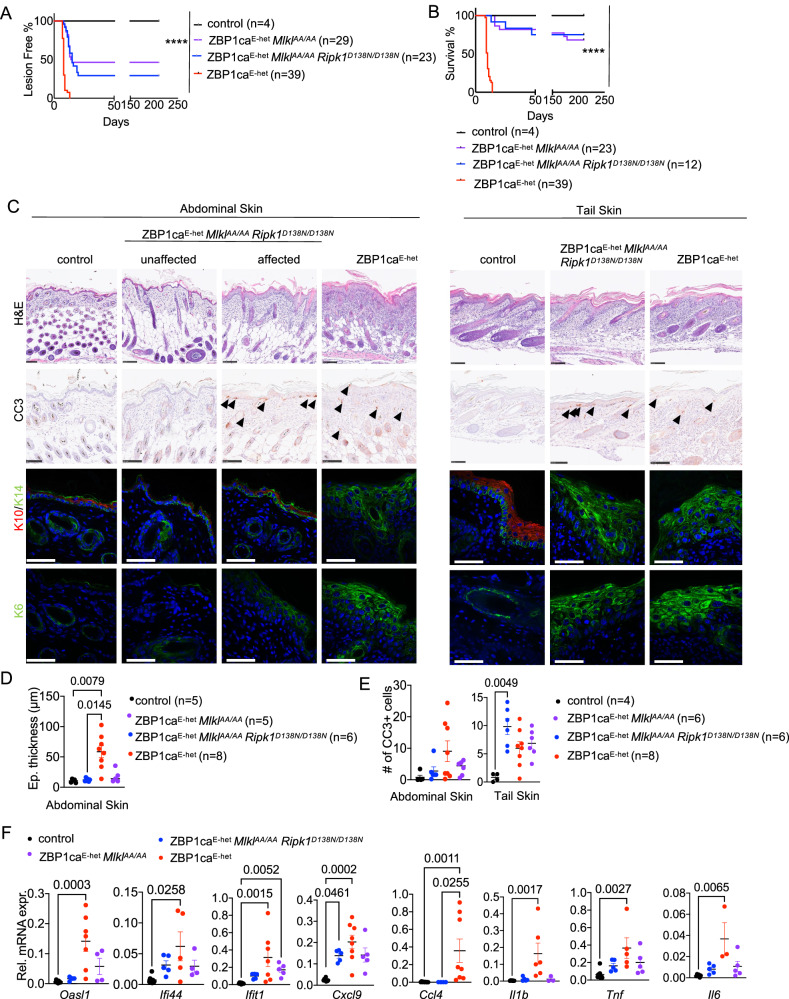
RIPK1 causes MLKL-independent skin inflammation in ZBP1ca^E-het^ mice independently of its kinase activity. **A** Graph depicting lesion-free survival of ZBP1ca^E-het^, ZBP1ca *Mlkl*^*AA/AA*^, ZBP1ca^E-het^ *Mlkl*^*AA/AA*^ *Ripk1*^*D138N/D138N*^ mice and non-affected littermates. **B** Graph depicting survival curve of ZBP1ca^E-het^, ZBP1ca *Mlkl*^*AA/AA*^, ZBP1ca^E-het^ *Mlkl*^*AA/AA*^ *Ripk1*^*D138N/D138N*^ mice and non-affected littermates. **C** Representative images from skin sections of ZBP1ca^E-het^ and ZBP1ca^E-het^ *Mlkl*^*AA/AA*^*Ripk1*^*D138N/D138N*^ mice and control mice stained with H&E (Scale bars = 100 µM, *n* = 5 for ZBP1ca^E-het^ *Mlkl*^*AA/AA*^*Ripk1*^*D138N/D138N*^, *n* = 11 for ZBP1ca^E-het^, *n* = 5 for control), CC3^+^ (*n* = 5 for ZBP1ca^E-het^ *Mlkl*^*AA/AA*^*Ripk1*^*D138N/D138N*^, *n* = 5 for ZBP1ca^E-het^, *n* = 5 for control), or immunostained with K10, K14 and K6 (Scale bars = 50 µM, *n* = 5 for ZBP1ca^E-het^ *Mlkl*^*AA/AA*^ *Ripk1*^*D138N/D138N*^, *n* = 8 for ZBP1ca^E-het^, *n* = 5 for control) at P10–12. Data from the same ZBP1ca^E-het^ and ZBP1ca^E-het^ *Mlkl*^*AA/AA*^ mice are included in all Figures for comparison. **D** Graph depicting epidermal thickness of mice with indicated genotypes at P10–12. Mean ± SEM are shown. Each dot represents one mouse. Data from the same ZBP1ca^E-het^ and ZBP1ca^E-het^ *Mlkl*^*AA/AA*^ mice are included in all Figures for comparison. **E** Graph showing the amount of CC3^+^ cells in abdominal and tail skin of mice with indicated genotypes. Mean ± SEM are shown. Each dot represents one mouse. **F** Graphs depicting relative mRNA expression of the indicated ISGs and cytokines in RNA from whole-skin tissue of P10–12 mice of the indicated genotypes measured by qRT-PCR. Each dot represents one mouse. Mean ± SEM are shown. Data from the same ZBP1ca^E-het^ and ZBP1ca^E-het^ *Mlkl*^*AA/AA*^ mice are included in all Figures for comparison. Statistical significance was determined using Kruskal–Wallis test (one-sided) in (**D**–**F**).

Our results above showed that ZBP1ca activates RIPK1 in a RHIM-dependent manner to induce caspase-8-mediated apoptosis in keratinocytes. In TNFR1 signaling, RIPK1 induces caspase-8-mediated apoptosis in a kinase activity-dependent manner. We therefore postulated that ZBP1ca may drive keratinocyte apoptosis by triggering RIPK1-kinase-dependent activation of caspase-8 in keratinocytes. To address the role of RIPK1 kinase activity we employed *Ripk1*^*D138N/D138N*^ mice, which express kinase inactive RIPK1 [24], to generate ZBP1ca^E-het^ *Mlkl*^*AA/AA*^ *Ripk1*^*D138N/D138N*^ mice. Surprisingly, we found that ZBP1ca^E-het^ *Mlkl*^*AA/AA*^ *Ripk1*^*D138N/D138N*^ mice developed inflammatory skin lesions, in contrast to ZBP1ca^E-het^ *Mlkl*^*AA/AA*^ *Ripk1*^*mR/mR*^ mice that did not show skin inflammation. Macroscopic examination revealed that 16 out of 23 ZBP1ca^E-het^ *Mlkl*^*AA/AA*^ *Ripk1*^*D138N/D138N*^ mice developed skin lesions during the first 3 weeks of life. Most of these lesions were very mild, with 9 out of 12 mice surviving without signs of severe skin inflammation up to at least 30 weeks of age (Fig. 8A, B). Histological analysis showed that most areas of abdominal skin in ZBP1ca^E-het^ *Mlkl*^*AA/AA*^ *Ripk1*^*D138N/D138N*^ mice were unaffected, with only focal areas exhibiting epidermal thickening and upregulation of K14 and K6 expression (Fig. 8C, D). Abdominal skin areas exhibiting epidermal thickening showed increased numbers of CC3^+^ cells, which however were significantly reduced compared to ZBP1ca^E-het^ mice (Fig. 8E). Similar to ZBP1ca^E-het^ *Mlkl*^*AA/AA*^ mice, CC3^+^ cells were increased in all tail skin sections of the ZBP1ca^E-het^ *Mlkl*^*AA/AA*^ *Ripk1*^*D138N/D138N*^ mice observed (Fig. 8C, E). Moreover, the expression of inflammatory cytokines, chemokines and ISGs was ameliorated in ZBP1ca^E-het^ *Mlkl*^*AA/AA*^ *Ripk1*^*D138N/D138N*^ mice compared to ZBP1ca^E-het^ mice (Fig. 8F). Collectively, these results showed that RIPK1 kinase activity is not required for ZBP1ca-induced caspase-8-dependent keratinocyte apoptosis.

## Discussion

Regulation of cell death signaling in keratinocytes has emerged as a critical mechanism controlling skin homeostasis and inflammation, however, the specific functions of different cell death pathways and upstream receptors have remained poorly understood [32]. Here we identified a critical interplay between RIPK3-MLKL-dependent necroptosis and FADD-caspase-8-mediated apoptosis that controls skin inflammation downstream of TNFR1 and ZBP1. Our genetic studies showed that epidermis-specific ablation of FADD or caspase-8 caused skin inflammation by inducing RIPK3-MLKL-dependent necroptosis of keratinocytes. Our earlier studies revealed that TNFR1 deficiency delayed and ameliorated but did not fully prevent skin lesion development in FADD^E-KO^ mice [21], suggesting that TNFR1-independent mechanism cause keratinocyte necroptosis and inflammation in these mice. Here we show that combined ablation of TNFR1 and ZBP1 fully prevented skin inflammation in FADD^E-KO^ mice, demonstrating that ZBP1-mediated necroptosis drives TNFR1-independent skin inflammation upon keratinocyte-specific inhibition of FADD/caspase-8 signaling. Notably, ZBP1 ablation on its own was not sufficient to inhibit skin inflammation in FADD^E-KO^ mice [6]. Therefore, TNFR1-induced necroptosis drives the early lesions, while ZBP1 is activated later to induce TNFR1-independent keratinocyte necroptosis and inflammation in FADD^E-KO^ mice. This interplay is reminiscent of the role of TNFR1 and ZBP1 in FADD^IEC-KO^ mice, where they cooperate to induce intestinal inflammation [11]. Collectively, these studies identified ZBP1-mediated necroptosis as a potent trigger of TNFR1-independent inflammation in epithelial barrier tissues, suggesting that ZBP1 may be implicated in the pathogenesis of inflammatory diseases of the skin and the gut, particularly in patients that do not respond to anti-TNF therapy.

Our findings in FADD^E-KO^ mice, together with our earlier studies in RIPK1^E-KO^ and *Ripk1*^*mR/mR*^ mice, showed that ZBP1 induces inflammation by triggering RIPK3-MLKL-dependent necroptosis [6, 10, 11]. However, in these mouse models cells were sensitized to necroptosis by the loss of FADD or RIPK1 and were unable to undergo RIPK1-mediated caspase-8-induced apoptosis. Therefore, it remains unclear whether ZBP1 could also directly engage and activate RIPK1 to induce caspase-8-mediated apoptosis and associated pathology in vivo. Addressing this question was hampered by the fact that expression of ZBP1 does not induce death in wild-type cells, unless if these are sensitized by inhibition of caspase-8 [13]. Here we identified a C-terminally truncated version of ZBP1, which we termed ZBP1ca, that induces cell death in cells with intact RIPK1 and caspase-8 signaling. Doxycycline-inducible expression of ZBP1ca, but not full-length ZBP1, caused cell death in iMEFs by inducing both RIPK3-dependent necroptosis and caspase-8-mediated apoptosis. Moreover, ZBP1ca interacted with both RIPK3 and RIPK1, in contrast to full-length ZBP1 that co-immunoprecipitated with RIPK3 but not with RIPK1. These findings indicated that ZBP1ca could induce caspase-8-dependent apoptosis by directly interacting with and activating RIPK1. In addition, these studies suggested that the C-terminal part of ZBP1 containing the second and third RHIMs inhibits ZBP1-induced cell death, consistent with previous studies showing that ZBP1-deficient cells reconstituted with constructs expressing ZBP1 with mutated RHIM2 or truncation of the C-terminal part of the protein containing RHIM3 underwent increased death in response to viral infection [33].

Our in vivo genetic studies showed that ZBP1ca expression induced keratinocyte death and caused skin inflammation. Mutation of the RIPK3 RHIM or the RIPK3 phosphorylation sites in MLKL strongly suppressed but could not fully prevent skin lesion development in ZBP1ca^E-het^ mice, showing that ZBP1ca induces skin inflammation primarily by triggering RIPK3-MLKL-mediated keratinocyte necroptosis and, to a lesser extent, by necroptosis-independent mechanisms. Caspase-8 ablation combined with mutation of the MLKL phosphorylation sites completely prevented skin lesion development in ZBP1ca^E-het^ mice, demonstrating that ZBP1ca induces skin inflammation by triggering necroptosis and caspase-8-mediated apoptosis. Mutation of the RIPK1 RHIM combined with mutation of the MLKL phosphorylation sites fully prevented skin lesion development, showing that ZBP1ca induced keratinocyte apoptosis by engaging RIPK1. Surprisingly, we found that, in contrast to mutation of its RHIM, inhibition of the kinase activity of RIPK1 together with mutation of the MLKL phosphorylation sites did not prevent caspase-8-mediated inflammation in ZBP1ca^E-het^ mice, arguing that ZBP1ca induces caspase-8-mediated apoptosis in a RIPK1 kinase activity-independent manner. These results are reminiscent of the RIPK1 kinase activity-independent caspase-8-mediated apoptosis induced after inhibition of RIPK3 kinase activity, either genetically by mutation of aspartic acid in position 161 to asparagine (D161N) [34] or in response to high concentration of RIPK3 inhibitors [35]. Therefore, while its kinase activity is required for inducing apoptosis downstream of TNFR1, RIPK1 induces caspase-8 activation in a kinase-independent manner downstream of RIPK3 and ZBP1. Although the underlying molecular mechanism remains to be fully elucidated, these results suggest that when RIPK1 is engaged via its death domain its kinase activation and autophosphorylation are required for the formation and activation of the death-inducing signaling complex resulting in the activation of caspase-8. In contrast, when RIPK1 is engaged via its RHIM downstream of ZBP1 or RIPK3, then it induces caspase-8 activation in a kinase-independent manner. This finding is relevant for the treatment of disease as it implies that RIPK1 kinase inhibitors may not be effective in suppressing RIPK1-mediated apoptosis and inflammation downstream of ZBP1 activation.

ZBP1 has been suggested to induce inflammatory gene expression independently from cell death [4, 5, 29, 36]. However, we did not observe upregulation of NF-κB-dependent gene expression in response to doxycycline-induced overexpression of FL-ZBP1 or ZBP1ca in iMEFs. Moreover, ZBP1ca^E-het^ mice did not show increased expression of cytokines and ISGs in the skin at P2, arguing that ZBP1ca does not directly drive inflammatory gene expression. Importantly, ZBP1ca^E-het^ mice showed upregulation of inflammatory genes in the skin at P10, which were strongly suppressed by inhibition of necroptosis and fully normalized by combined ablation of necroptosis and apoptosis. Therefore, in this in vivo experimental system ZBP1ca drives cytokine production indirectly by inducing RIPK3-MLKL-dependent necroptosis and to a lesser extent by RIPK1-caspase-8-mediated apoptosis. Our interpretation of these results is that cytokine production is not directly caused by ZBP1ca but is induced by the release of DAMPs from the dying cells. Interestingly, ZBP1ca additionally induced the expression of ISGs, which also depended largely on necroptosis as they were strongly suppressed by mutation of the RIPK3 RHIM or the MLKL phosphorylation sites. Combined mutation of the RIPK1 RHIM and the MLKL phosphorylation sites fully normalized ISG expression in ZBP1ca^E-het^ mice. However, ZBP1ca^E-het^ *Mlkl*^*AA/AA*^ *Casp8*^E-KO^ mice showed strongly enhanced ISG expression compared to ZBP1ca^E-het^ mice, although cytokine expression levels were fully normalized in these animals. While the underlying mechanism remains elusive at present, these results suggest that caspase-8 has an important role in suppressing ISG expression in the skin of ZBP1ca^E-het^ mice.

Collectively, we identified ZBP1 as a critical factor that cooperates with TNFR1 to induce cell death and inflammation. ZBP1 utilizes its first RHIM to interact with RIPK3 and RIPK1 inducing necroptosis and apoptosis respectively, whereas its C-terminal part encompassing the second and third RHIMs appears to exert inhibitory functions that prevent ZBP1-mediated cell death. Importantly, our results provided in vivo experimental evidence that ZBP1 causes inflammation by triggering necroptosis and, to a lesser extent, apoptosis, arguing against a role of ZBP1 in inducing cell death-independent inflammatory signaling. These findings suggest that ZBP1 could be implicated in the pathogenesis of inflammatory diseases, particularly in patients that do not respond to anti-TNF therapy.

### Supplementary information

Supplementary Figures

## Data Availability

The datasets generated during and/or analyzed during the current study are available from the corresponding author on reasonable request.
