# Supplementary material for: ZBP1 causes inflammation by inducing RIPK3-mediated necroptosis and RIPK1 kinase activity-independent apoptosis
Source: Cell Death Differ. 2024 Jun 7;31(7):938–53. doi: 10.1038/s41418-024-01321-6 (PMC11239871; doi:10.1038/s41418-024-01321-6)
Supplement: Supplementary file 1 — Supplementary Figures [file 41418_2024_1321_MOESM1_ESM.pdf]

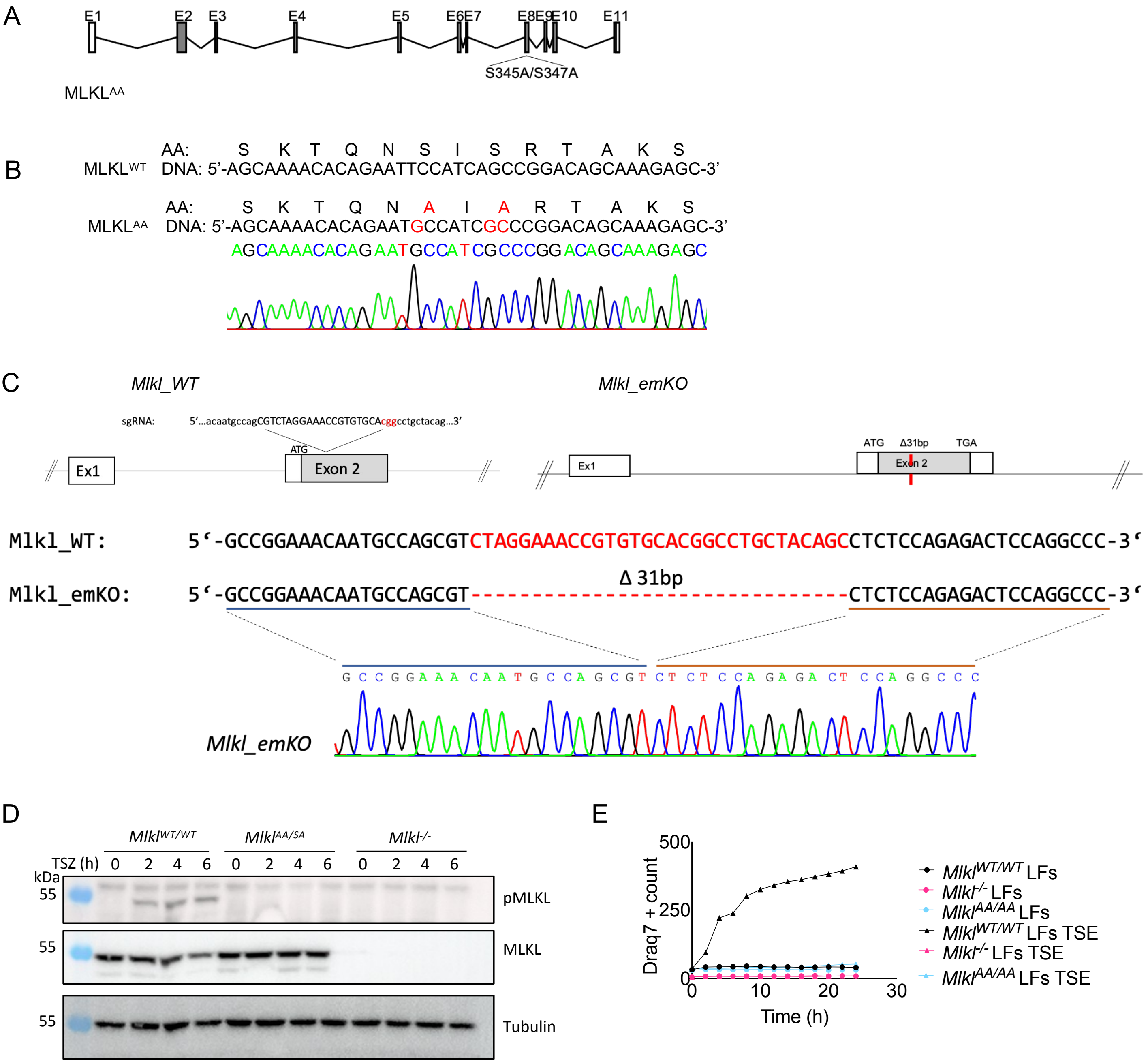

Supplementary Figure 1. Generation of *Mlkl*$^{AA/AA}$ and *Mlkl*$^{-/-}$ mice

(A) Serines at positions 345 and 347 encoded from exon 8 in the mouse *Mlkl* gene were mutated to alanines using CRISPR/Cas9-mediated gene targeting in zygotes from C57BL/6N mice, using the gRNA target sequence 5' AAACACAGAATTCCATCAGC3' and the ssODN repair template 5' AACGTCTGCATCTAACACATCTGTCTGTCTAGCTTGCAGGATTTGAGTTAAGCAAAACACAGAAT GCCATCGCCCGGACAGCAAAGAGCACTAAAGCAGAGAGATCCAGTTCAACGATATATGTCTCCCCT GAGAGAC3' to generate the *Mlkl*^{AA} mice. **(B)** Successful generation of the *Mlkl*^{AA} allele was assessed by Sanger sequencing of genomic DNA; red nucleotides (DNA) and amino acids (Prot.) indicate the desired mutations that are visible on the sequencing trace from a homozygous *Mlkl*$^{AA/AA}$ mouse. **(C)** The *Mlkl*$^{-}$ allele was generated using CRISPR/Cas9-mediated gene targeting in zygotes from C57BL/6N mice, using a single sgRNA binding in exon 2 of the mouse *Mlkl* gene. The *Mlkl*$^{-}$ allele resulted from a 31 bp deletion (visible in the Sanger sequencing trace from genomic DNA of a homozygous mouse) that caused a frameshift and premature stop codon (TGA) in exon 2. **(D)** Expression of total MLKL and phosphorylated MLKL (pMLKL) protein was assessed by western blot after TNF (T), Smac mimetic (S) and Z-VAD-FMK (Z) stimulation with tubulin as a loading control. **(E)** Incucyte based live imaging analysis of cell death in *Mlkl*$^{WT/WT}$, *Mlkl*$^{-/-}$ and *Mlkl*$^{AA/AA}$ cells upon stimulation with TNF (T), Smac mimetic (S) and Emricasan, with total count of dead cells (Draq7+) displayed over time.

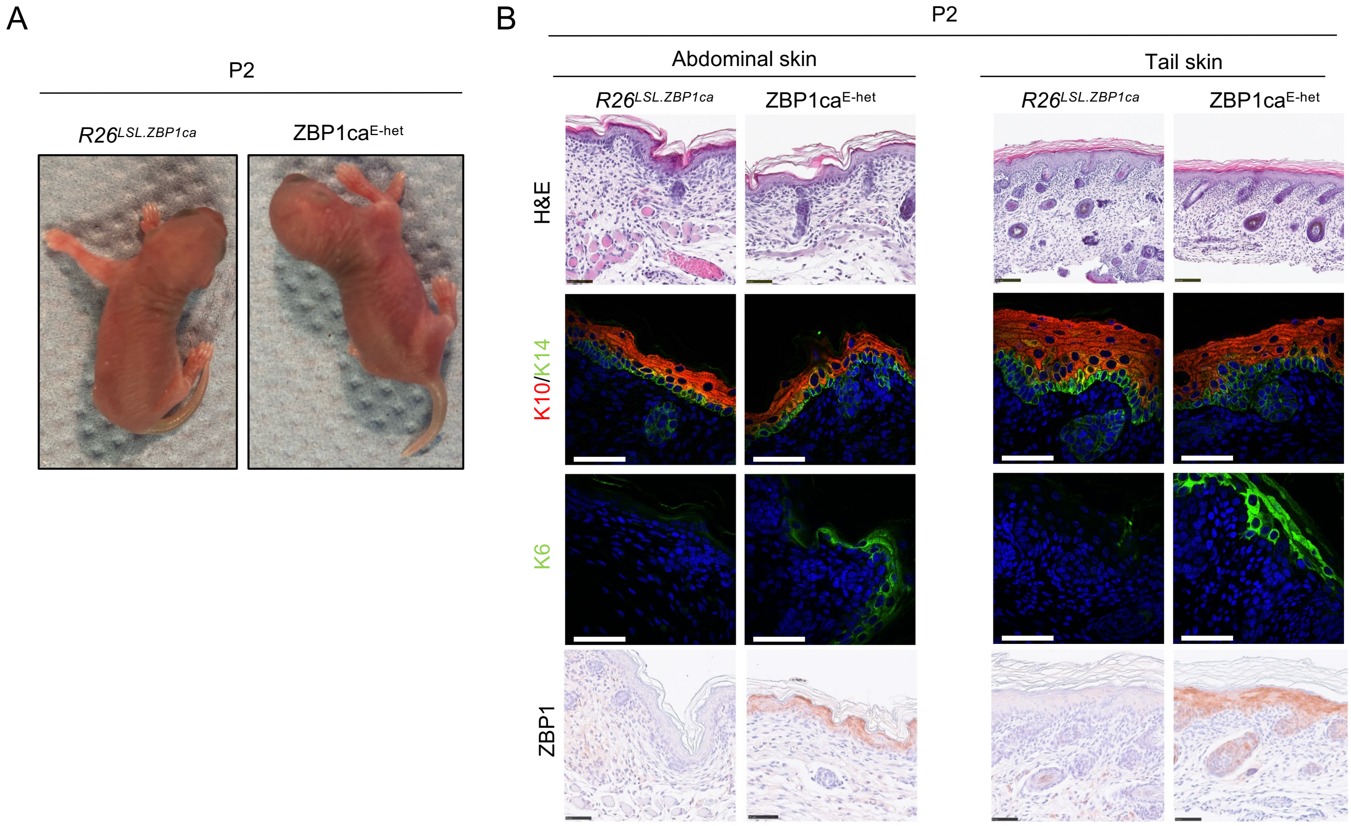

Supplementary Figure 2. Normal skin structure and upregulation of K6 in the epidermis of ZBP1ca$^{E\text{-}het}$ mice at postnatal day 2.

(A) Representative photographs of ZBP1ca$^{E\text{-}het}$ mouse (n=7) and control littermate (n=12) at P2. **(B)** Representative images from skin sections of ZBP1ca$^{E\text{-}het}$ and *R26*$^{LSL.ZBP1ca}$ littermates at P2 stained with H&E (Scale bars=50 µM, n=6 for ZBP1ca$^{E\text{-}het}$, n=5 for control), or immunostained for ZBP1 (Scale Bars=50 µM, n=4 for ZBP1ca$^{E\text{-}het}$ n=2 for control), K10, K14, K6 (Scale bars=50 µM, n=4 for ZBP1ca$^{E\text{-}het}$, n=4 for *R26*$^{LSL.ZBP1ca}$).

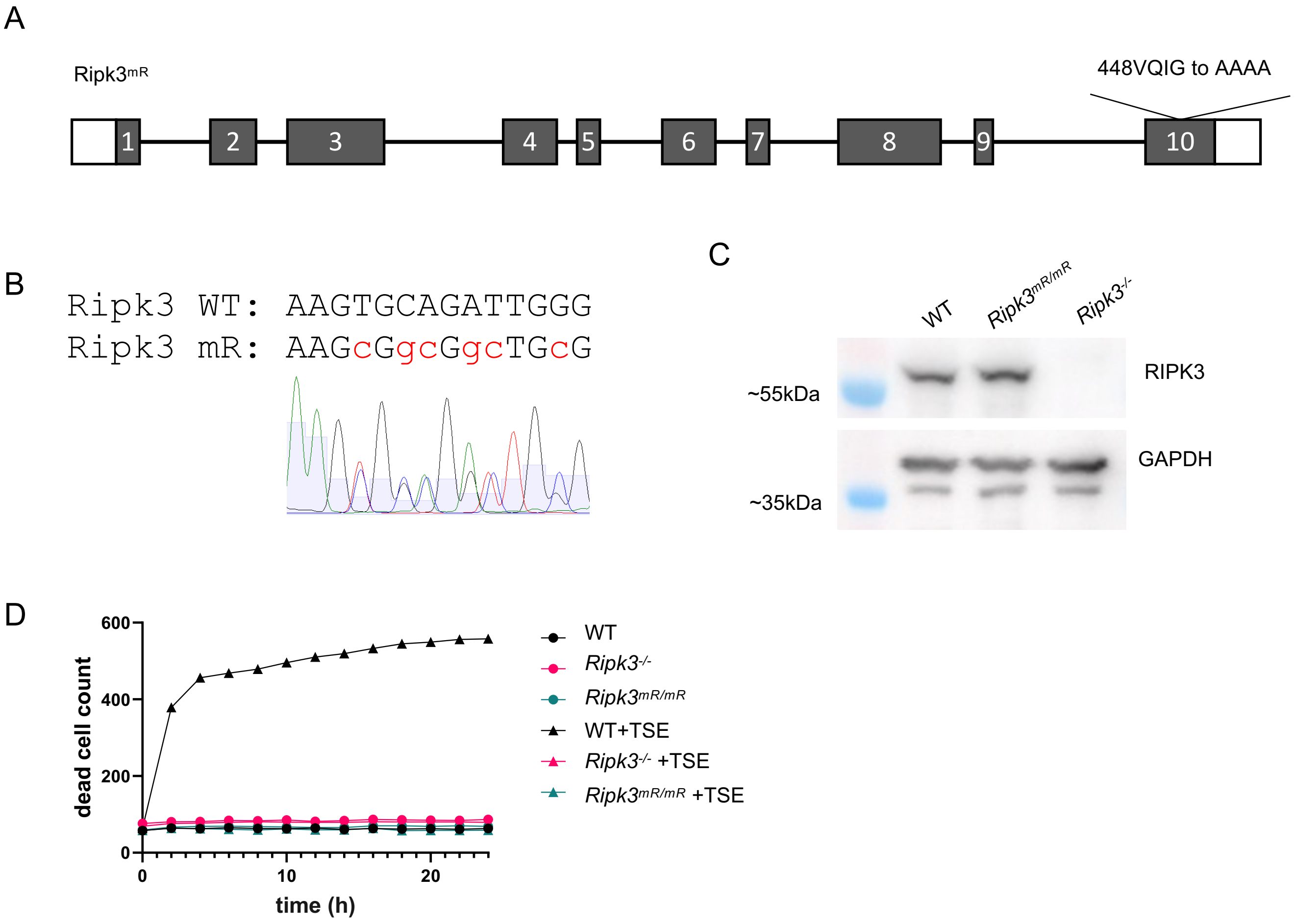

Supplementary Figure 3. Generation and characterization of *Ripk3*$^{mR/mR}$ mice.

(A) The core RHIM motif in exon 10 of the *Ripk3* gene was altered from the amino acid sequence VQIG to AAAA using CRISPR/Cas9-mediated gene targeting in C56BL/6 mouse zygotes using the gRNA target sequence 5'ACTGTTCTGAAGTGCAGATT3' and the repair ssODN 5'CACCATCACCTCCTCC TTTCCTCTTAAAGGGCCACCGGCTCTCGTCTTCAACAACTGTTCTGAAGCGGCGGCTGCGAACTACA ACTCCTTGGTAGCACCACCAAGAACTACTGCCTCAAGTTCGGCCAAGTATGACCAAG3'. **(B)** Sanger sequencing of genomic DNA indicates the desired mutations in heterozygous *Ripk3*$^{mR/WT}$ mice with the mutated nucleotides indicated in red. **(C)** Immunoblot analysis of primary lung fibroblasts (LFs) with *Ripk3*$^{-/-}$ cells as a control **(D)** Incucyte-based life cell imaging of cell death in primary LFs from mice with the indicated genotypes stimulated with TNF (T), Smac mimetic (S) and Emricasan (E) (TSE treatment) revealed that *Ripk3*$^{mR/mR}$ cells are resistant necroptosis similarly to *Ripk3*$^{-/-}$ LFs

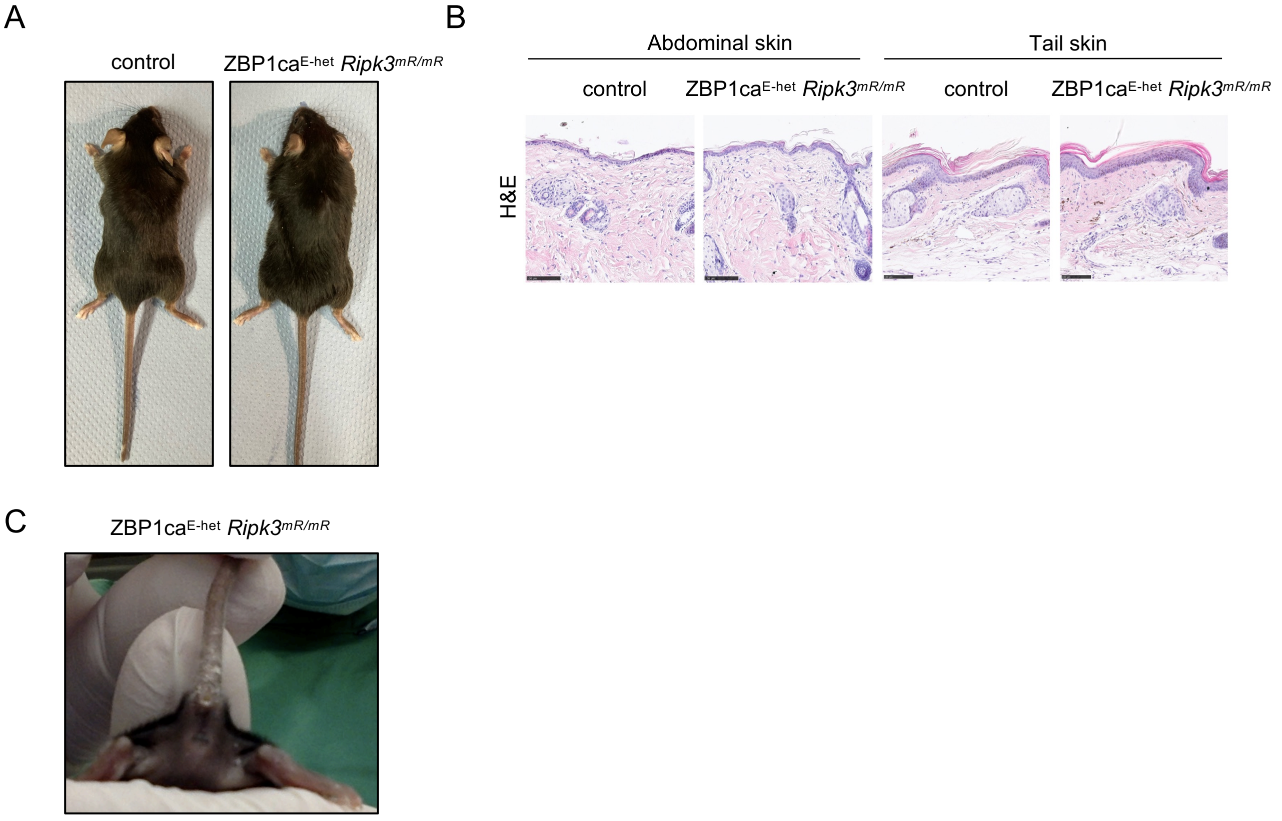

Supplementary Figure 4. Macroscopic and histological analysis of ZBP1ca$^{E\text{-}het}$ *Ripk3*$^{mR/mR}$ mice.
(A) Representative photographs of ZBP1ca$^{E\text{-}het}$ *Ripk3*$^{mR/mR}$ mouse (n=12) and control (n=3) at 30 weeks of age. **(B)** Representative images from skin sections of ZBP1ca$^{E\text{-}het}$ *Ripk3*$^{mR/mR}$ mice and littermate at 30 weeks of age stained with H&E (Scale bars=100 µM, n=6 for ZBP1ca$^{E\text{-}het}$ *Ripk3*$^{mR/mR}$, n=3 for control). **(C)** Representative photograph of ZBP1ca$^{E\text{-}het}$ *Ripk3*$^{mR/mR}$ mouse at P14.

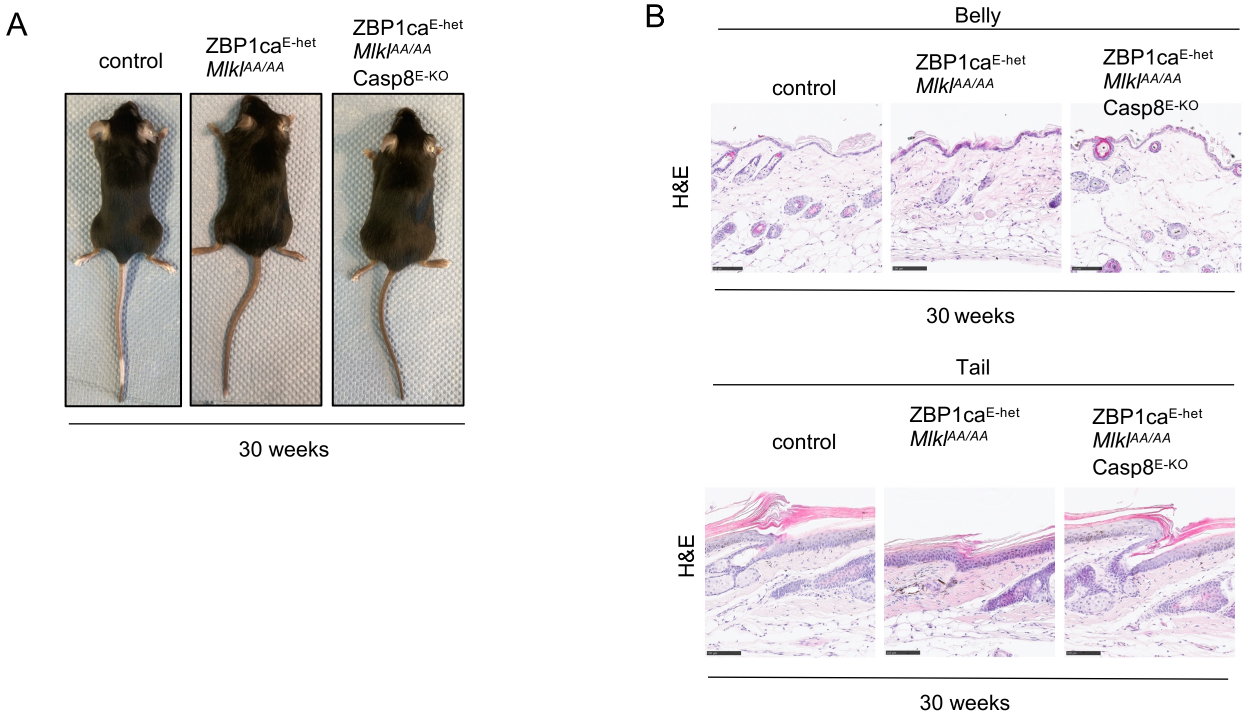

Supplementary Figure 5. Macroscopic and histological analysis of ZBP1ca$^{\text{E-het}}$ *Mlkl*$^{AA/AA}$ and ZBP1ca *Mlkl*$^{AA/AA}$ Casp8$^{\text{E-KO}}$ mice at 30 weeks of age.

(A) Representative photographs of ZBP1ca$^{\text{E-het}}$ *Mlkl*$^{AA/AA}$ (n=12), ZBP1ca$^{\text{E-het}}$ *Mlkl*$^{AA/AA}$ Casp8$^{\text{E-KO}}$ (n=13) and control (n=13) at 30 weeks of age). **(B)** Representative images from abdominal and tail skin sections of ZBP1ca$^{\text{E-het}}$ *Mlkl*$^{AA/AA}$, ZBP1ca$^{\text{E-het}}$ *Mlkl*$^{AA/AA}$ Casp8$^{\text{E-KO}}$ mice and control at 30 weeks of age stained with H&E (Scale bars=100 μM, n=5 for ZBP1ca$^{\text{E-het}}$ *Mlkl*$^{AA/AA}$, n=3 for ZBP1ca$^{\text{E-het}}$ *Mlkl*$^{AA/AA}$ Casp8$^{\text{E-KO}}$, n=4 for control).

Figure 3

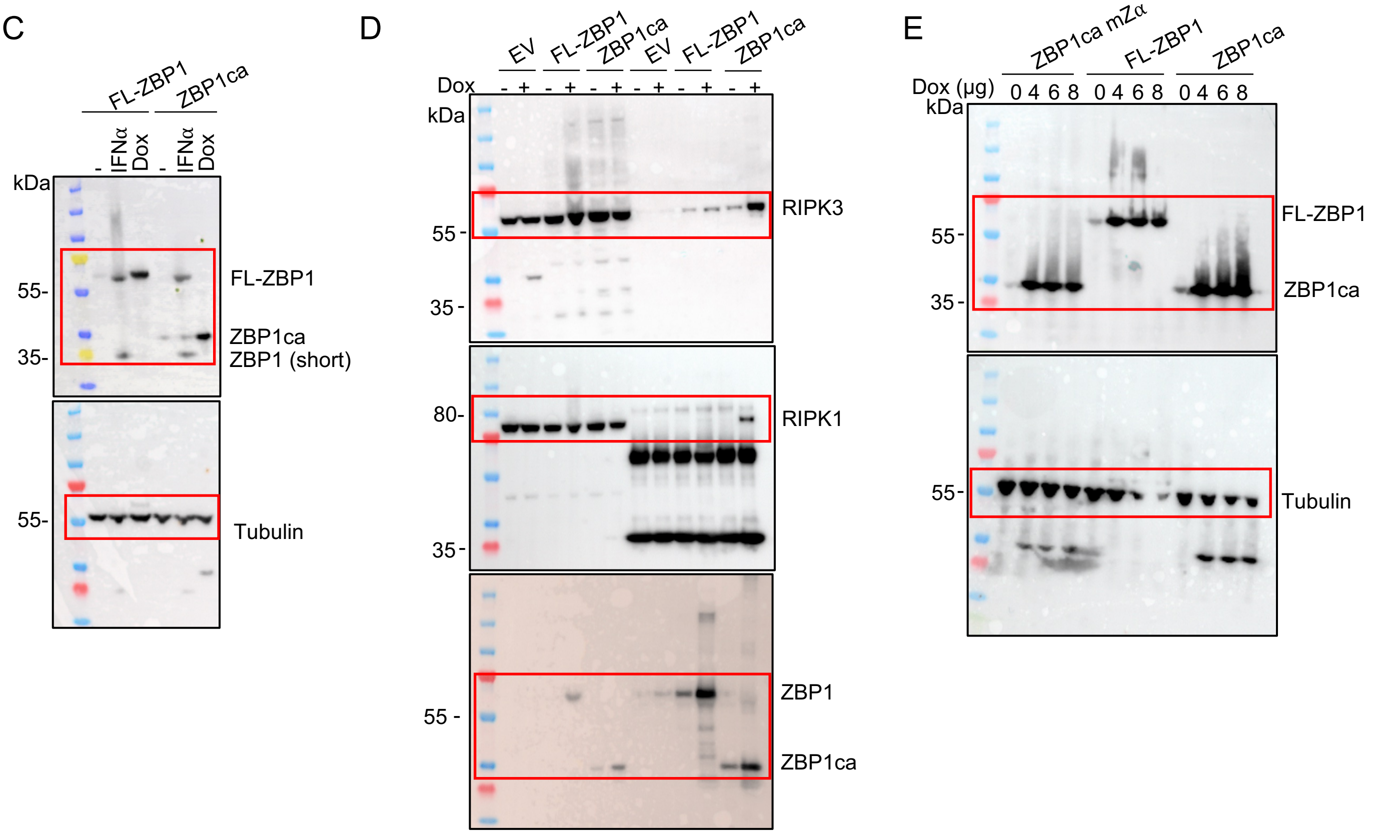

Figure 4

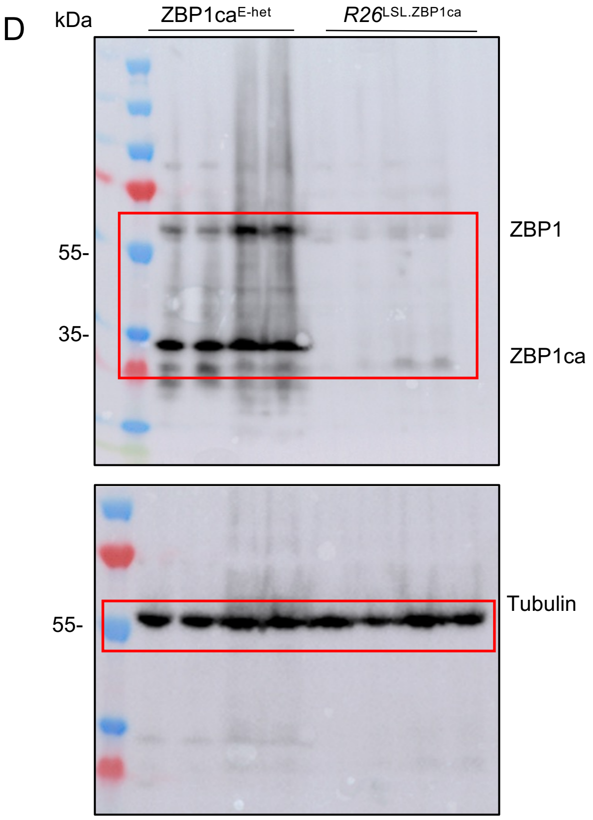

Supplementary Figure 6. Uncropped gels of immunoblots presented in Figures 3 and 4.
